# Enhancing the Output Performance of Tubular Gas‐Liquid Mixing Triboelectric Nanogenerator by Bulk Effect

**DOI:** 10.1002/advs.202510787

**Published:** 2025-08-22

**Authors:** Yang Dong, Jiahui Cheng, Zhichen Cao, Nannan Wang, Di Yang, Suping Chang, Wenlong Lu

**Affiliations:** ^1^ School of Mechanical Science and Engineering Huazhong University of Science and Technology Wuhan 430074 P. R. China; ^2^ State Key Laboratory of Intelligent Manufacturing Equipment and Technology Huazhong University of Science and Technology Wuhan Hubei 430074 P. R. China; ^3^ State Key Laboratory of Solid Lubrication Lanzhou Institute of Chemical Physics Chinese Academy of Sciences Lanzhou 730000 P. R. China; ^4^ HUST‐Shenzhen Research Institute Shenzhen 518000 P. R. China

**Keywords:** bulk effect, energy harvesting, gas‐liquid mixing flow, triboelectric nanogenerator

## Abstract

Gas‐liquid two‐phase flow‐based triboelectric nanogenerators (GL‐TENGs) have gained widespread attention due to their ability to convert the kinetic energy of complex flowing fluids into electrical power, but limitation such as relatively low output power density imposed by interfacial effects severely restrict their output performance. Here, a novel tubular bulk effect gas‐liquid mixing triboelectric nanogenerator (TBE‐GL‐TENG) is designed to significantly enhance the output performance of GL‐TENGs. The instantaneous output voltage, output current, and transferred charge of 1530 V, 112 µA, and 0.33 µC are obtained for a TBE‐GL‐TENG device when used with 1.0 mL of tap water, which are ≈5.3, 9.6, and 3.0 times higher than those of the control group based on conventional interfacial effects, respectively. With an instantaneous power output of 18.67 kW m^−3^, 640 LEDs can be directly powered by a TBE‐GL‐TENG device. The working mechanism of TBE‐GL‐TENG is systematically elucidated from microscopic simulations to macroscopic experiments, and the key factors for enhancing instantaneous output power are discussed in detail. This proposed tubular bulk effect electricity generator based on gas‐liquid mixing flow will open new insight in water energy harvesting and self‐powered sensing for multi‐phase flow systems.

## Graphical abstract

A novel tubular bulk effect gas‐liquid mixing triboelectric nanogenerator (TBE‐GL‐TENG) is designed to address the relatively low output power density imposed by the traditional interface effect. The introduction of the electrode into the PTFE tube successfully couples the bulk effect with the gas‐liquid mixing flow system, significantly improving the instantaneous output performance of GL‐TENGs.

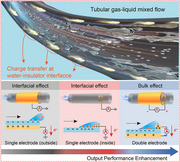

## 1 Introduction

With the rapid development of the Internet of Things (IoT), distributed sensing, and ocean energy development, the need for sustainable micro‐energy supply technologies is becoming increasingly urgent.^[^ ^1^, ^2^, ^3^ ^]^ Various energy harvesting technologies such as photovoltaic,^[^ ^4^, ^5^ ^]^ piezoelectric,^[^ ^6^, ^7^ ^]^ and thermoelectric^[^ ^8^, ^9^ ^]^ are still difficult to harvest low‐frequency, widely distributed, and irregular energy. Triboelectric nanogenerators (TENGs), as an energy conversion technology based on the coupling of contact electrification and electrostatic induction, have emerged as a promising solution for converting ubiquitous mechanical energy into electrical power.^[^ ^10^, ^11^, ^12^, ^13^, ^14^ ^]^ Considering their advantages of simple structure, wide choice of materials, and suitability for large‐scale diffusion, TENGs have attracted much attention worldwide since it was first invented by Prof Wang's team in 2012.^[^ ^15^ ^]^ Among them, the solid‐liquid TENGs (SL‐TENGs) can effectively harvest energy from water by inducing charge transfer through the dynamic contact‐separation process between liquid and solid dielectric materials. In 2013, Lin et al. innovatively demonstrated a water‐TENG based on the contact electrification between a patterned polydimethylsiloxane (PDMS) pyramid array film and water for harvesting liquid‐wave energy.^[^ ^16^ ^]^ The water‐TENG can obtain an open‐circuit voltage of 52 V and a short‐circuit current density of 2.45 mA m^−2^, and the output can instantaneously light up 60 LEDs. Subsequently, more and more researchers have improved the output performance of SL‐TENGs through superhydrophobic nanostructures and functional group surface modification of the contact materials, gradually opening a new chapter of SL‐TENGs for harvesting energy from water in the forms of raindrops,^[^ ^17^, ^18^ ^]^ river and ocean waves,^[^ ^19^, ^20^ ^]^ and others.^[^ ^21^, ^22^ ^]^ However, the low surface charge transfer efficiency imposed by conventional interfacial effects remains a challenge that limits the output performance of SL‐TENGs.

Remarkably, a transistor‐inspired droplet‐based electricity generator (DEG) was innovatively proposed to circumvent the limitations imposed by interfacial effects, thus significantly improving the instantaneous output performance of SL‐TENGs.^[^ ^23^ ^]^ The core innovation of the DEG device is that the spreading of the droplet bridges disconnected components into a closed‐loop electrical system and transforms the conventional interfacial effect into the desirable so‐called “bulk effect.” Building on this pioneering work, researchers have boosted the output performance of the solid‐liquid electricity generators and expanded their application scenarios through connecting electrodes design, dielectric material selection, and intermediate dielectric layers.^[^ ^24^, ^25^, ^26^, ^27^, ^28^, ^29^, ^30^ ^]^ However, SL‐TENGs mostly rely on the mechanical action of single‐phase fluid (e.g., droplets or waves), and the small contact area and the low contact separation speed during solid‐liquid contact electrification severely limit their output performance.^[^ ^31^, ^32^ ^]^ Considering the complex interfacial dynamics behavior of tubular high‐speed gas‐liquid mixing flow, combining it with TENG technology provides new ideas to address the above challenges. For example, the turbulence‐enhancing effect of the fluid can increase the solid‐liquid contact electrification area, while the curvature effect of the tube wall may induce localized charge aggregation and directional transport. Representatively, Dong et al. developed a gas‐liquid two‐phase flow‐base TENG (GL‐TENG) with a Venturi‐like fluid structure, which combined contact electrification and breakdown effect to enable several order‐of‐magnitude increase in the instantaneous output power of the SL‐TENGs.^[^ ^33^ ^]^ Subsequently, the GL‐TENGs have been widely studied as an emerging energy harvesting strategy and have achieved good results.^[^ ^34^, ^35^ ^]^ However, it remains a challenge to overcome the interfacial effects to construct an efficient and stable energy conversion system from fluid kinetic energy to triboelectric power.

Herein, a novel tubular bulk effect gas‐liquid mixing triboelectric nanogenerator (TBE‐GL‐TENG) with high output performance was designed. The bulk effect was successfully coupled into the tubular gas‐liquid mixing flow regime by the design of dual electrodes inside and outside a 6‐cm‐long polytetrafluoroethylene (PTFF) tube. Using 1 mL of tap water, a peak open‐circuit voltage of 1530 V and a peak short‐circuit current of 112 µA were obtained by a TBE‐GL‐TENG device at a specific gas‐liquid mixed‐flow pulse frequency. The output performance has good stability and can directly light up 640 commercial light‐emitting diodes (LEDs) by leveraging the instantaneous output power of 18.67 kW m^−3^. Moreover, the working mechanism of the TBE‐GL‐TENG has been systematically elucidated in terms of simulation, experiment, and circuit, respectively, and the key factors to enhance the instantaneous output power were discussed in detail. The results of this study lay a theoretical and technological foundation for accelerating the development of a new generation of high‐performance in SL‐TENGs.

## 2 Results and Discussion

### 2.1 Design and Output Performance of TBE‐GL‐TENG

**Figure 1:**
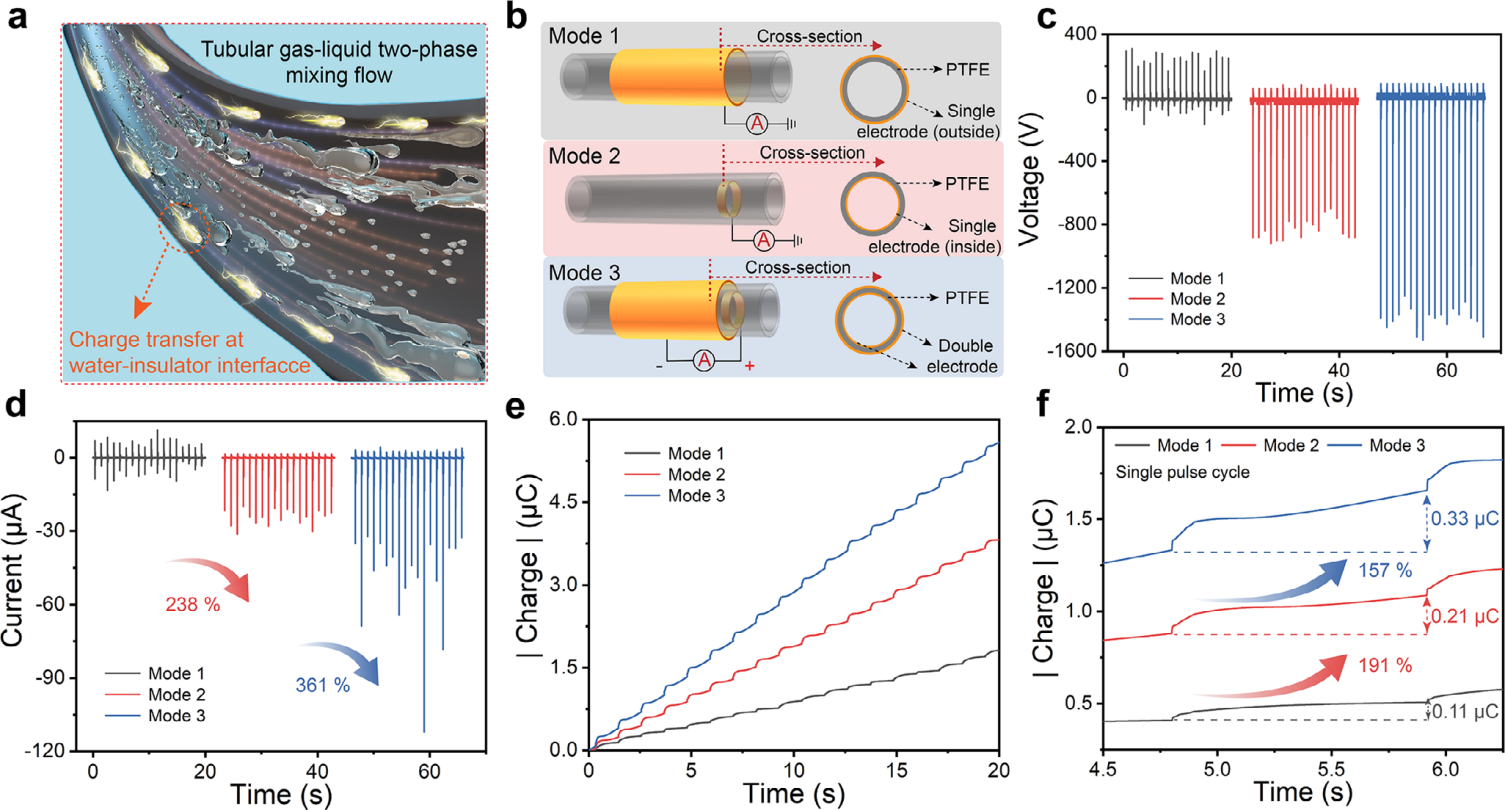
Design and performance of the TBE‐GL‐TENG. a) Schematic of charge transfer at the water‐insulator interface in a tubular gas‐liquid mixed flow. b) Structural design of three‐mode GL‐TENG. “Mode 1” and “Mode 2” are based on conventional interfacial effect while “Mode 3” is based on bulk effect. Comparison of output voltage c), current d), and transferred charge e) between three modes of GL‐TENGs. f) Comparison of the transferred charge from “Mode 3” (in blue), control device “Mode 2” (in red), and control device “Mode 1” (in black) in response to 1.0 mL tap water flow through the three electricity generators.

The high velocity flow of a tubular gas‐liquid two‐phase stream will generate huge kinetic energy, which can be converted into electrical power by the triboelectric effect at the interface between the liquid and the insulator. As shown in **Figure** **1**a, friction between the water and the insulator surface induces charge transfer and electrostatic buildup, and this charge can be used directly for power generation through the design of an external circuit. Our proposed TBE‐GL‐TENG mainly consists of a 6.0‐cm‐long and 1.0‐mm‐thick PTFE tube, an annular electrode attached to the outer wall as the negative electrode, an annular electrode fixed to the inner wall as the positive electrode, and some connecting wires. Noted that the TBE‐GL‐TENG device is also referred to as “Mode 3” generator throughout the text (Figure 1b). Meanwhile, two GL‐TENGs based on conventional interfacial effects were prepared as the control groups. They are single‐electrode GL‐TENGs obtained by designing electrodes on the outside (Noted as “Mode 1”) and inside (Noted as “Mode 2”) of a 6.0‐cm‐long and 1.0‐mm‐thick PTFE tube, respectively. Subsequently, each of the three modes of electricity generators was mounted in the middle of an over‐flow pipe so that the gas‐liquid mixed flow runs through them to achieve water energy harvesting. A multiphase flow system connected to the inlet of the overflow pipe can generate a gas‐liquid two‐phase mixed flow with controlled pressure, flow rate, and frequency. The inlet pressure of the gas flow was maintained at 0.6 MPa, and the velocity of the gas‐liquid mixing flow could be up to 30.0 m s^−1^. During the test, the gas‐liquid mixing flow was maintained at a typical pulse frequency with a continuous jet‐on time of 1.0 s and a jet‐off time of 0.1 s, and a single pulse cycle containing 1.0 mL of water. If not otherwise specified, tap water was used for testing considering that it is cheap and readily available. Compared with triboelectricity at the water/PTFE interface, the triboelectricity at the gas/PTFE interface can be neglected (Figure S1, Supporting Information). Focusing on an individual “Mode 3” generator indicates that the open‐circuit output voltage and short‐circuit current were ≈1530 V (Figure 1c) and 112 µA (Figure 1d). And the peak voltage were ≈5.3 and 1.7 times higher than the values obtained from “Mode 1” and “Mode 2,” while the peak current were ≈9.6 and 3.6 times higher than the values obtained from “Mode 1” and “Mode 2,” respectively. As shown in Figure 1e, an integral analysis of the short‐circuit current over eighteen cycles revealed that the transferred charge of the “Mode 3” generator was ≈5.6 µC, which was much higher than that of “Mode 2” (≈3.8 µC) and “Mode 1” (≈1.8 µC). Careful inspection of the transferred charge during a single output cycle indicated that “Mode 3” transferred a much higher amount of charge then the “Mode 1” and “Mode 2” under the same circumstances (Figure 1f). In addition, this “bulk effect” enhancement of transferred charge can be more intuitively demonstrated through one full current cycle and corresponding charge accumulation curve (Figure S2, Supporting Information). Compared with similar work reported previously, this TBE‐GL‐TENG performs exceptionally well (See Table S1, Supporting Information).

### 2.2 Charge Transfer Mechanisms at Water‐Insulator Interfaces

**Figure 2:**
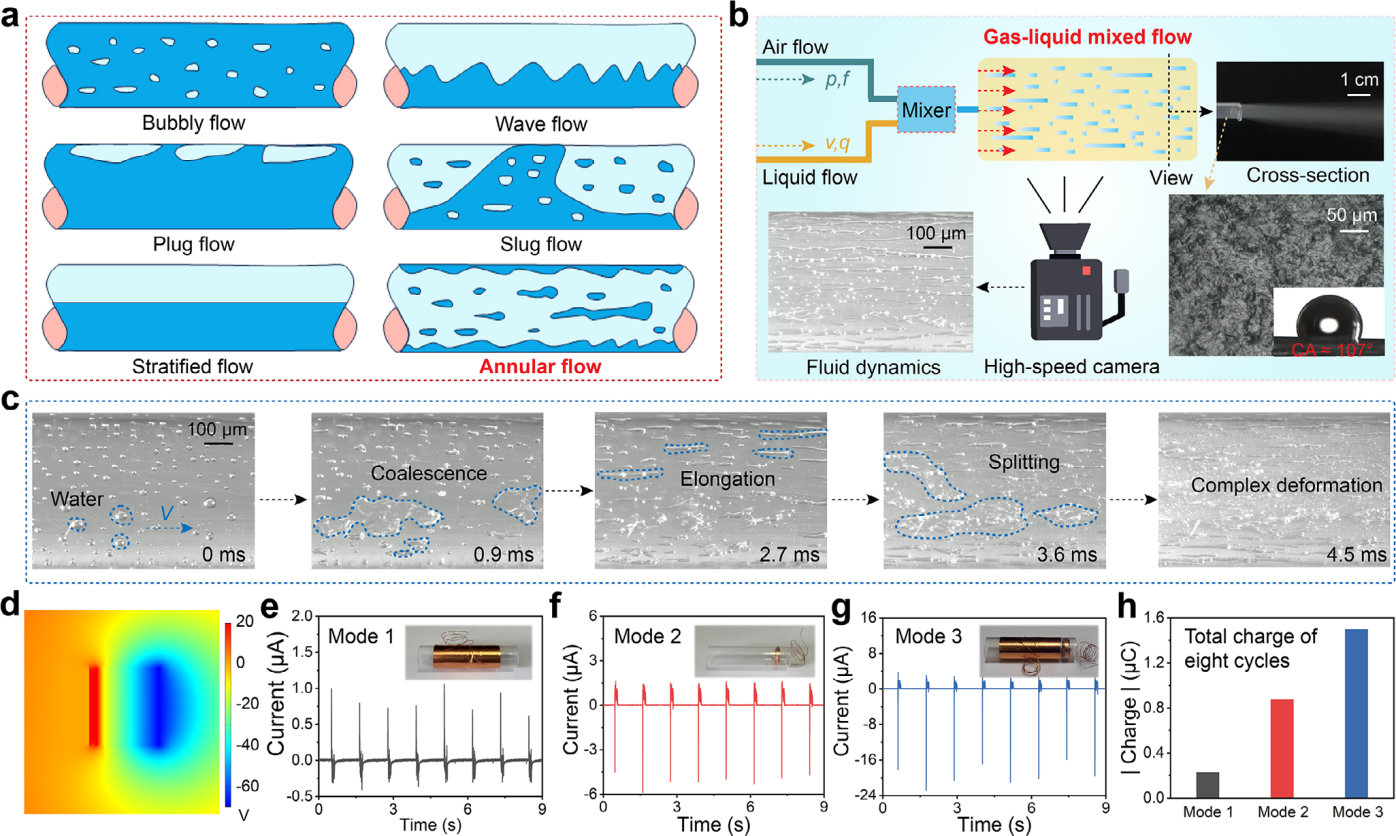
Charge transfer dynamics at water‐insulator interfaces. a) Classification of gas‐liquid mixed flow patterns in a horizontal pipe. b) Dynamical observation of horizontal annular flow with a high‐speed camera. c) Complex dynamics of liquids on the inner wall of the insulator, including coalescence, elongation, and splitting. d) Voltage simulation between water and PTFE in contact. e–g) Output currents of the three power generation modes when all other conditions are held constant. h) The transferred charges in the three modes show that “Mode 3” is superior to “Mode 2” and “Mode 1.”.

The boost in the output performance of the “Mode 3” device compared with the conventional design suggests that the TBE‐GL‐TENG device might operate via a different mechanism. In order to deeply investigate the charge transfer mechanism of TBE‐GL‐TENG, the kinetic behavior of the tubular gas‐liquid mixing flow was first analyzed. As shown in **Figure** **2**a, the gas‐liquid two‐phase mixed flow in a horizontal pipe can be classified into six flow patterns according to the flow velocity, pressure, and mass vapor content. And they are bubbly flow, plug flow, stratified flow, wave flow, slug flow, and annular flow. The flow pattern transition of gas‐liquid two‐phase mixed flow in a horizontal pipe has a significant impact on the output performance of TBE‐GL‐TENG. The core lies in the fact that flow pattern changes directly regulate fluid dynamics characteristics and water/PTFE interface contact behavior. In order to reduce the impact of gas‐liquid two‐phase mixed flow patterns on TBE‐GL‐TENG output performance, the gas pressure was kept constant at 0.6 MPa throughout the experiment to keep the flow pattern as constant as possible. Considering the fact that most of the liquid actually moves along the wall of the PTFE tube as an annular film, while the gas flows at high speed in the center region of the tube entrapped in a mist of foam. It is clear that the current flow pattern of the gas‐liquid mixing flow is annular flow. As shown in Figure 2b, a gas‐phase flow with controllable pressure and frequency, and a liquid‐phase flow with controllable velocity and flow rate were coupled in a mixer to form an annular flow with controllable kinematic parameters. In particular, the liquid phase control system includes a high‐precision digital display peristaltic pump that can adjust the output flow range (0–90 mL min^−1^) in real time according to requirements. By combining the gas phase system, controlling the frequency of the gas flow allows for producing a quantitative volume of liquid. The top right inset was a physical view of a cross‐section of a gas‐liquid mixed flow running pipe, showing that most of the liquid exhibits an annular liquid film moving along the pipe wall, which is consistent with the previous annular flow analysis. And the lower right inset was an electron micrograph of the PTFE surface, which shows a uniform compositional distribution of its components. The static contact angle between the insulator PTFE and water was ≈107°, and the hydrophobic property will reduce the liquid adhesion and accelerate the solid‐liquid contact separation process. The dynamics of the moving liquids inside the tube were recorded by a high‐speed camera (Revealer) at a typical recording speed of 40 000 frames per second (Video S1, Supporting Information).

As shown in Figure 2c, the motion state of water in a gas‐liquid mixing flow undergoes a complex change in a very short period of time. The shear force of the high‐speed air flow overcomes the surface tension of the liquid and tears the liquid into numerous small droplets (atomization). And many broken small droplets may also merge or break up further after the collision. For example, the region marked by a blue dashed in Figure 2c, the droplets undergo changes such as coalescence, elongation, and splitting, ultimately forming a two‐phase mixing flow with complex motions. And this complex motion is also accompanied by contact electrification between the liquid and the PTFE surface. Affected by the solid‐liquid contact area, contact separation frequency, and other factors, the charge transfer behavior between water and PTFE will change dramatically. Regardless of how the amount of charge transfer changes, it can be determined that PTFE is negatively charged due to the strong electron‐withdrawing effect, while the water loses electrons and becomes positively charged.^[^ ^36^, ^37^ ^]^ This is proven by the results of the voltage simulation of the contact between water and PTFE (Figure 2d). Subsequently, the three modes of electricity generators were tested with deionized water, and the peak output short‐circuit currents obtained were ≈1.0 µA (Figure 2e), −6.0 µA (Figure 2f), and −24.0 µA (Figure 2g), respectively. And the insets in the current output graphs were the physical diagrams of the corresponding generators for the three modes. Further integral analysis of the currents over eight cycles indicated that the transferred charge of “Mode 3” was also the largest among the three modes of solid‐liquid electricity generators (Figure 2h).

**Figure 3:**
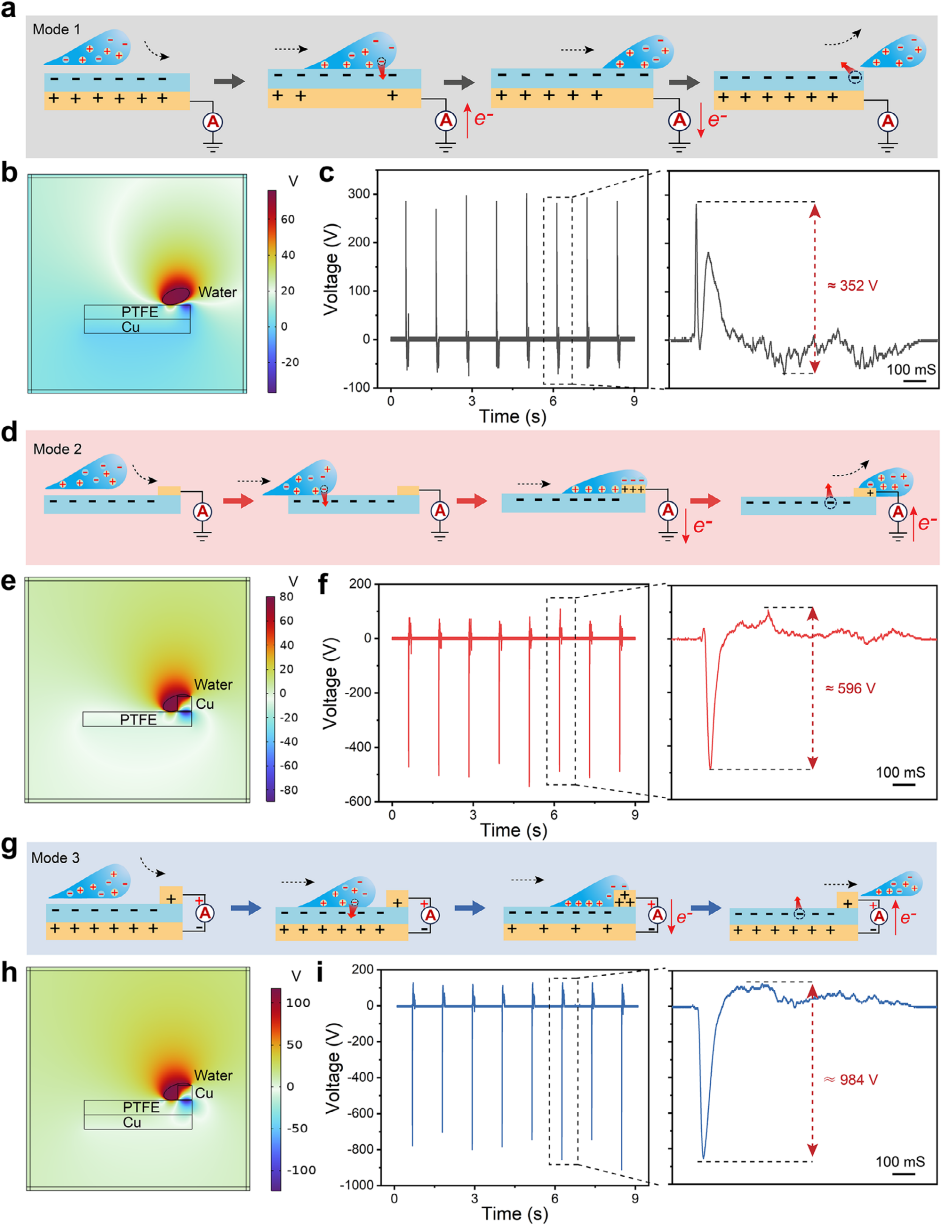
Mechanism of charge transfer in the three power generation modes. a) Schematic drawing showing the process of electricity generation for “Mode 1.” b) COMSOL Multiphysics simulation of the surface potential distribution of the “Mode 1” electricity generator with bottom electrode. c) Typical voltage pattern of “Mode 1.” The right panel is the enlarged view of the region marked by a black dashed rectangle. d) Schematic drawing showing the process of electricity generation for “Mode 2.” e) COMSOL Multiphysics simulation of the surface potential distribution of the “Mode 2” electricity generator with top electrode. f) Typical voltage pattern of “Mode 2.” The right panel is the enlarged view of the region marked by a black dashed rectangle. g) Schematic drawing showing the process of electricity generation for “Mode 3.” h) COMSOL Multiphysics simulation of the surface potential distribution of the “Mode 3” electricity generator with bulk effect. i) Typical voltage pattern of “Mode 3.” The right panel is the enlarged view of the region marked by a black dashed rectangle.

The charge transferred mechanism of the GL‐TENGs is closely related to the dynamics of a substantial number of droplets in a gas‐liquid two‐phase mixed flow. In order to illustrate the charge transfer mechanism of GL‐TENGs, an individual droplet is selected as a research object to simplify their complex power generation process. As shown in **Figure** **3**a, the power generation process of “Mode 1” with a single electrode at the bottom indicates that the transferred charge originates from the triboelectricity at the water‐PTFE interface. Before droplet impact, the negatively charged PTFE induces an equal positive charge on the grounded Cu electrode. As the droplet spreads over the PTFE surface, the contact electrification between the water and PTFE results in charge transfer. The result is that the water loses electrons and becomes positively charged while the PTFE gains electrons and becomes negatively charged (Figure S3, Supporting Information). And the substantial negative charges on the PTFE surface will attract the positive charge in the water, which in turn form an electrostatic shield on the PTFE surface. The existence of an electrostatic effect disrupts the charge balance between the PTFE and the grounded Cu electrode, and a positive charge of the electrode transfers to ground rapidly, thus an instantaneous output is generated in the external circuit. During droplet leaving the PTFE surface, the positive charge gradually flows back to the electrode from the ground. When the droplet leaves completely, some of the negative charge on the PTFE surface dissipates into the surrounding environment. The charge balance between the PTFE and the electrode is re‐established and ready for the next power generation. To further elucidate this charge transfer mechanism, COMSOL Multiphysics software was used to simulate the surface potential distribution of the contact between water and PTFE. As shown in Figure 3b, the simulation result of “Mode 1” device shows that the water is positively charged while the PTFE is negatively charged, which is consistent with the previous analysis. Details of the “Mode1” finite element simulation of geometric modeling and mesh generation are shown in Figure S4 (Supporting Information). The working mechanism is proven by the time‐resolved voltage waveform in Figure 3c, in which the typical voltage output peaks approaching 352 V.

Similar to the working mechanism of the “Mode 1” device, the source of charge transfer in the “Mode 2” device is also the triboelectricity between the water and PTFE interface. As shown in Figure 3d, the PTFE is pre‐charged due to contact electrification. When a neutral droplet contacts the negatively charged PTFE surface, the positive charge in the water is attracted by the negative charge on the surface. And the strong mutual attraction between the dissimilar charges causes the positive charge in the water to be tightly packed on the PTFE surface. Once the moving droplet contacts the grounded Cu electrode, an electrical connection is established between the PTFE and the electrode. As the negative charge in the water flows toward the electrode, the imbalance in potential between PTFE, water, and the electrode causes electrons to flow from the electrode to ground, thus generating a high instantaneous output in the external circuit. When the droplet leaves the electrode, electrons flow back from the ground to the Cu electrode thus re‐establishing the potential balance of the whole system. This entire power generation process is accompanied by contact electrification between the water and the PTFE, as well as dissipation of the charge on the PTFE into the surrounding environment. Simulation of the surface potential distribution by COMSOL Multiphysics software shows that there is a significant potential difference between the water, PTFE, and electrode (Figure 3e; Figure S5, Supporting Information). And this potential difference is the root cause of the high instantaneous output of the “Mode 2” device. This working mechanism is also evidenced the time‐resolved voltage waveform, as shown in Figure 3f, with an instantaneous peak positive and negative output voltage difference of ≈596 V.

Significantly different from conventional “Mode 1” and “Mode 2” generator operation, the charge transfer in the TBE‐GL‐TENG originates from the prestored charge in the PTFE rather than triboelectricity between water and PTFE. The prestored charges are formed through the continuous flow of water droplets over the PTFE surface, which usually takes 10 cycles of a gas‐liquid mixing pulse cycle given that the charge density of the pristine PTFE is zero. As shown in Figure 3g, when a droplet contacts the top electrode, it acts as a bridge connecting the PTFE and the two electrodes to form a closed‐loop electrical system, and thus the power generation changes from conventional interface effect to bulk effect. Benefiting from the bulk effect, the sufficient charges prestored in the PTFE are rapidly transferred between the two electrodes, thus generating a high instantaneous electrical power output. Simulation of the surface potential shows a large potential difference between the water, PTFE, and electrodes, which also illustrates the superiority of this structural design (Figure 3h; Figure S6, Supporting Information). In terms of experimental results, the bulk effect power generation “Mode 3” with 1.0 mL of DI water can generate an instantaneous voltage output of ≈984 V, which is considerably higher than that of the conventional interface effect “Mode 1” and “Mode 2” generators (Figure 3i).

### 2.3 Mechanism of Bulk Effect to Enhance Output Performance

The working mechanism of TBE‐GL‐TENG can be better interpreted from the perspective of a circuit. Noted that GL‐TENG involves countless small droplets during the power generation process, and a single droplet is chosen as the object of analysis for a clearer understanding of its working mechanism. The electricity generation in the single‐electrode GL‐TENG with interface effect can be treated as the charge transfer between two capacitors (Figure S7, Supporting Information). When the moving droplet contacts the PTFE tube and the Cu electrode, an electric double layer (EDL) forms at the water/PTFE and water/Cu interfaces, respectively, which can be treated as capacitor *C*_EDL1_ and capacitor *C*_EDL2_. The resistances of the water and the load are *R*_W_ and *R*_L_, respectively. The core of power generation is that the triboelectricity at the water/PTFE interface breaks the potential equilibrium of the whole system, thus generating a directional movement of charge in the external circuit and forming an instantaneous electrical power output. This power generation process does not form a complete closed‐loop electrical system, and the output performance is limited by the interface effect.

**Figure 4:**
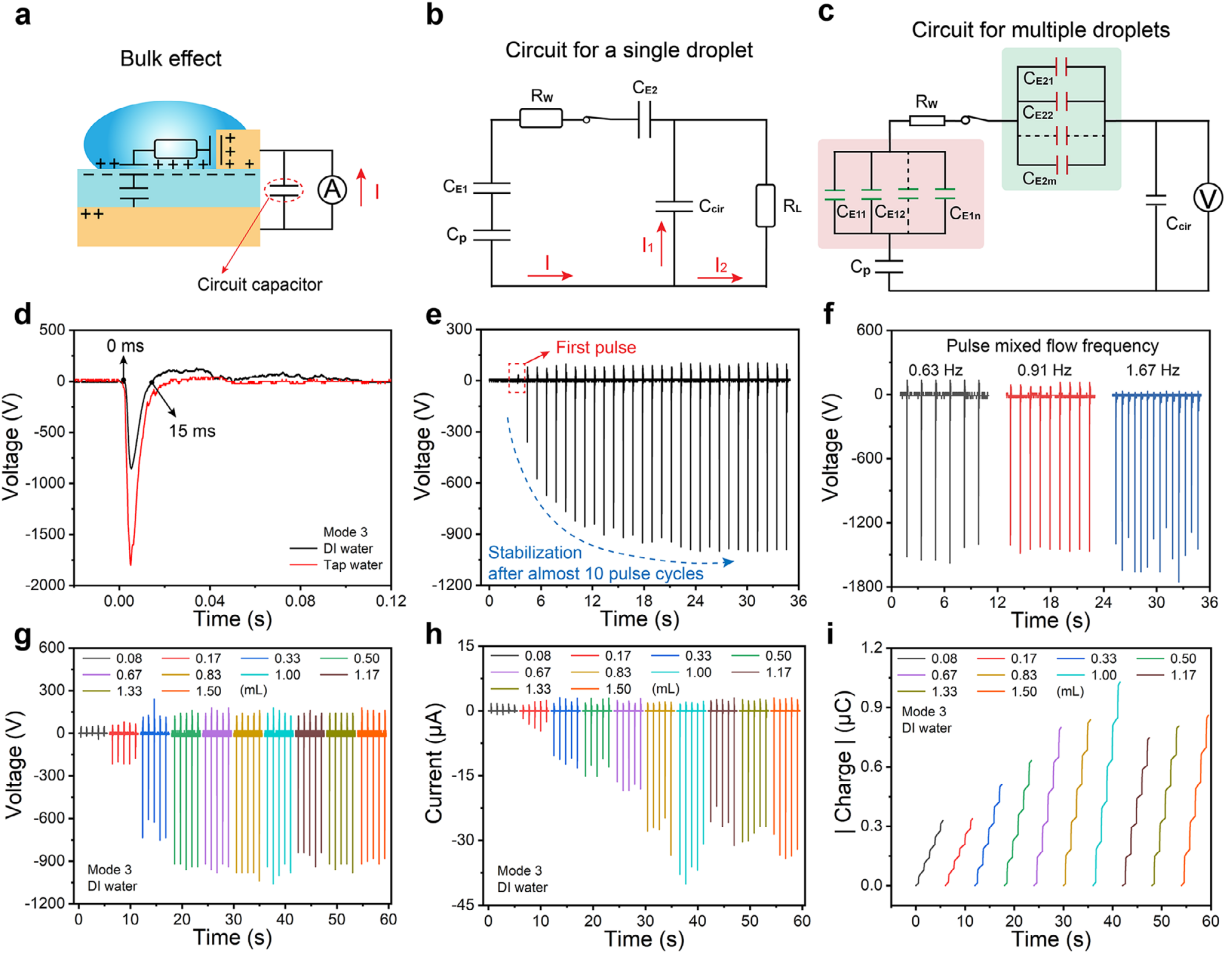
Working mechanism and circuit model for enhancing instantaneous output performance by the bulk effect. a) Design of a power generator with bulk effect (Mode 3). b) Established an equivalent circuit model for a single droplet. c) Established an equivalent circuit model for multiple droplets. d) Typical output voltage signals for a “Mode 3” device with DI water and tap water, respectively. e) Voltage stabilization. f) Voltage versus gas‐liquid two‐phase mixed flow pulse frequency. g–i) Output voltage, current, and transferred charge versus DI water volume.

In contrast to the above mechanism, the electricity generation in TBE‐GL‐TENG can be treated as the charge transfer between three capacitors (**Figure** **4**a). The first capacitor is *C*_P_, in which the water/PTFE serves as the top plate and the PTFE/Cu serves as the bottom plate. Considering the strong electron‐withdrawing effect and the strong charge‐storing ability of PTFE, the PTFE obtains sufficient negative charge from the continuously impinging droplets. Therefore, the PTFE can be regarded to be charge saturated before power generation. While the moving droplet contacts the PTFE tube, an EDL forms at the water/PTFE interface, which can be treated as the second capacitor, *C*_E1_. The impacting and flowing processes of the droplet convert the mechanical energy to electrical energy at the water/PTFE interface, which is equivalent to charging *C*_E1_. Subsequently, as the boundaries of the flowing droplet contacts the top Cu electrode, another EDL forms at the water/Cu interface, giving rise to the third capacitor, *C*_E2_. Considering that the thickness of the PTFE (1000 µm) is several orders of magnitude large than the Debye length of EDL (roughly 1 µm for deionized water)^[^ ^38^ ^]^ at the water/solid interfaces, the charge stored in *C*_p_ is dramatically higher than that stored in *C*_E1_ and *C*_E2_. As shown in Figure 4b, the introduction of *C*_E2_ bridges *C*_p_, *C*_E1_, *C*_E2_, *R*_W,_ and *R*_L_ to form a closed‐loop electrical system, where *R*_W_ and *R*_L_ are the resistances of the water and the load, respectively. In practical electrical measurements, there unavoidably and undesirably exists a circuit capacitor, *C*_cir_. And the adverse shunting effect introduced by the *C*_cir_ reduces charge release to the external load (see the shunting current *I*_1_ in Figure 4b). The droplet dynamically controls the on/off state of the whole power generation device, depending on its contact status with the top Cu electrode.

As shown in Figure 4c, the equivalent circuit involving multiple droplets is more complex. When multiple droplets contact the inner ring‐shaped Cu electrode simultaneously, multiple EDL capacitors, *C*_E21_, *C*_E22_, …, *C*_E2m_, are formed. Here, m represents the number of droplets simultaneously in contact with the electrode, which changes in real time with variations in fluid dynamics. Since these capacitors share a common plate (Cu), they can be equivalent to a parallel connection. Similarly, when multiple liquids come into contact with PTFE, multiple EDL capacitors *C*_E11_, *C*_E12_, …, *C*_E1n_ are formed. Here, n represents the number of droplets simultaneously in contact with the PTFE. For these capacitors share a common plate (PTFE), so they can be equivalent to a parallel connection. Considering the characteristics of parallel capacitors, multiple capacitors connected in parallel increase the total capacitance, thereby increasing the electrical output.

Considering the case of a single droplet, the introduction of *C*_E2_ bridges the circuit into the “switch‐on” mode, and the charge stored in *C*_p_ and *C*_E1_ is rapidly released to the capacitor *C*_E2_ thus generating an instantaneous electrical output as shown in Figure 4d. When deionized water was replaced with tap water, the peak output voltage of the TBE‐GL‐TENG increased notably from ≈−856 to ≈−1800 V. The enhancement of the peak voltage was attributed to the increase in ionic concentration of the liquid. Many studies have shown that the ion concentration significantly affects the magnitude of charge transferred at the solid‐liquid interfaces, which directly affects the output performance of the solid‐liquid electricity generators. Prior to the test, the PTFE tube in the TBE‐GL‐TENG was treated with an ion fan to bring the PTFE surface charge density to zero. As shown in Figure 4e, the peak value of the output voltage of the “Mode 3” device gradually increases and stabilizes after about ten pulse cycles, indicating that it takes only a very short time and a small amount of water to saturate the charge on the PTFE surface. And in the subsequent power generation process, the charging (the equivalent of contact electrification) at the water/PTFE interface and the discharging in the external circuit maintain a dynamic balance. Moreover, the output is insensitive to the gas‐liquid mixed flow pulse frequency (Figure 4f). The output open‐circuit voltage (Figure 4g), short‐circuit current (Figure 4h), and transferred charge (Figure 4i) of the “Mode 3” generator showed a tendency to increase and then stabilize as the volume of water in a single pulse cycle increase. In a tubular gas‐liquid mixing flow system, an appropriate increase in water volume increases the solid‐liquid contact electrification area and thus enhances the charge transfer. However, when the volume of water increases to a certain value, the transferred charge at the solid‐liquid interfaces does not change dramatically, and the output performance of the electricity generator remains relatively stable.

### 2.4 Various Factors Affecting the Output Performance of the TBE‐GL‐TENG

**Figure 5:**
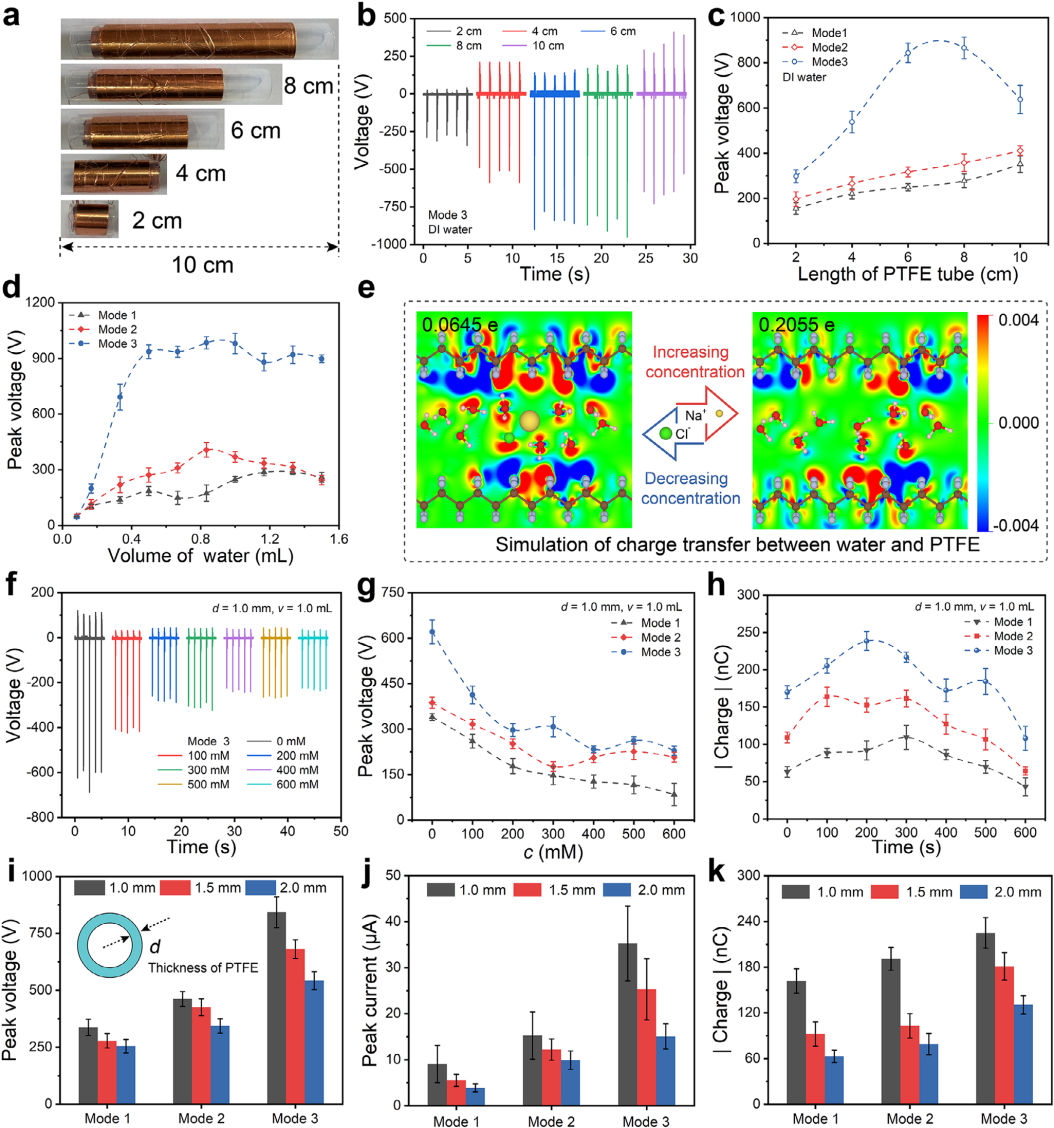
Broad impacts of the TBE‐GL‐TENG. a) Physical drawing of different lengths of PTFE tube. b) Output voltage versus PTFE length. c) Peak voltage as a function of PTFE tube length. d) Peak voltage as a function of water volume. e) Simulation of charge transfer between water and PTFE. f) Output voltage versus the concentration of NaCl solution. Peak voltage g) and transferred charge h) as a function of NaCl concentration. i–k) Peak voltage, peak current, and transferred charge versus thickness of PTFE tube.

Various factors affecting the output performance of TBE‐GL‐TENG were explored. As shown in **Figure** **5**a, PTFE tubes with a thickness of 1.0 mm were cut into lengths of 2, 4, 6, 8, and 10 cm to obtain the corresponding TBE‐GL‐TENGs. Subsequently, these five power generation devices were tested under the same conditions to obtain their electrical outputs. The experimental results show that the open‐circuit voltage exhibits a trend of increasing and then decreasing with the increase of PTFE length (Figure 5b). Benefiting from the contact electrification at the water/PTFE interface, the longer the PTFE tube is, the more charge it acquires from the water. However, considering the actual droplet slip length on the PTFE surface, the longer PTFE does not contribute to the closed‐loop electrical system for power generation. In contrast, the peak output voltages of “Mode 1” and “Mode 2” based on the interfacial effect increase with the increase of PTFE length (Figure 5c). Nevertheless, the output of “Mode 3” is much larger than that of “Mode 1” and “Mode 2” under the same conditions. The volume of the liquid also has a significant effect on the output performance of TBE‐GL‐TENG. As shown in Figure 5d, the peak output voltage increases and then stabilizes with the increase of droplet volume, and the two control generators show the same trend. And the relationship between electrical power output and liquid volume for “Mode 1” (Figure S8, Supporting Information) and “Mode 2” (Figure S9, Supporting Information) also exhibits the same trend as that for “Mode 3.”

Numerous studies have shown that triboelectricity at solid‐liquid interfaces involves both ion transfer and electron transfer.^[^ ^39^, ^40^, ^41^ ^]^ Therefore, the effects of ion concentration on the output performance of TBE‐GL‐TENG were systematically investigated from microscopic simulations and macroscopic tests. As shown in Figure 5e, a physical model of the corresponding water/PTFE interfacial contact was established, and the charge transfer at the water/PTFE interface was simulated based on density functional theory (DFT). The simulation results show that the number of Na^+^ and Cl^−^ in ten water molecules increases from 1 to 6, respectively, and the transferred charge increases from 0.0645 to 0.2055 e (For details, see Figure S10, Supporting Information). A moderate increase in the concentration of ions in the solution leads to an increase in the solid‐liquid interfacial charge transfer, which in turn increases the generator output performance. As shown in Figure 5f, with the increase of NaCl concentrations from 0 to 600 mm, the peak voltage decreases notably from 600 to 240 V. The ionic adsorption on the PTFE surface at higher ionic concentrations produces a “shielding effect,” which hinders the electron transfer process and leads to a reduction in electrical output. It is contributed to reduced Debye length and Debye time with the increase in ion concentrations, and the reduction in Debye length will intensify the “shielding effect.”^[^ ^42^, ^43^ ^]^ Further increasing the ion concentration would reduce the surface charge density on the PTFE tube, thereby reducing the electrical output.^[^ ^31^, ^41^ ^]^ The results with a series of NaCl solutions with a concentration gradient showed that the peak output voltage of the three modes of generators decreases with the increase of solution concentration (Figure 5g). However, the amount of transferred charge was not simply linear with NaCl concentration, but exhibited a trend of increasing and then decreasing. The experiment results in Figure 5h illustrate the complex effect of ion concentration on the output performance of TBE‐GL‐TENG. Finally, the effect of the thickness of the PTFE tube on the generator output performance is also explored. Comparison of the output open‐circuit voltage (Figure 5i), short‐circuit current (Figure 5j), and transferred charge (Figure 5k) analysis of the three modes of generators shows that their output performance is comprehensively reduced with the increase of PTFE thickness. The detailed relationship between the output performance of the “Mode 3” generator and the thickness of the PTFE tube is shown in Figure S11 (Supporting Information). Obviously, the electric output of the bulk effect “Mode 3” electricity generator is significantly higher than the interface effect “Mode 1” and “Mode 2” electricity generators under the same conditions.

### 2.5 Application of TBE‐GL‐TENG

**Figure 6:**
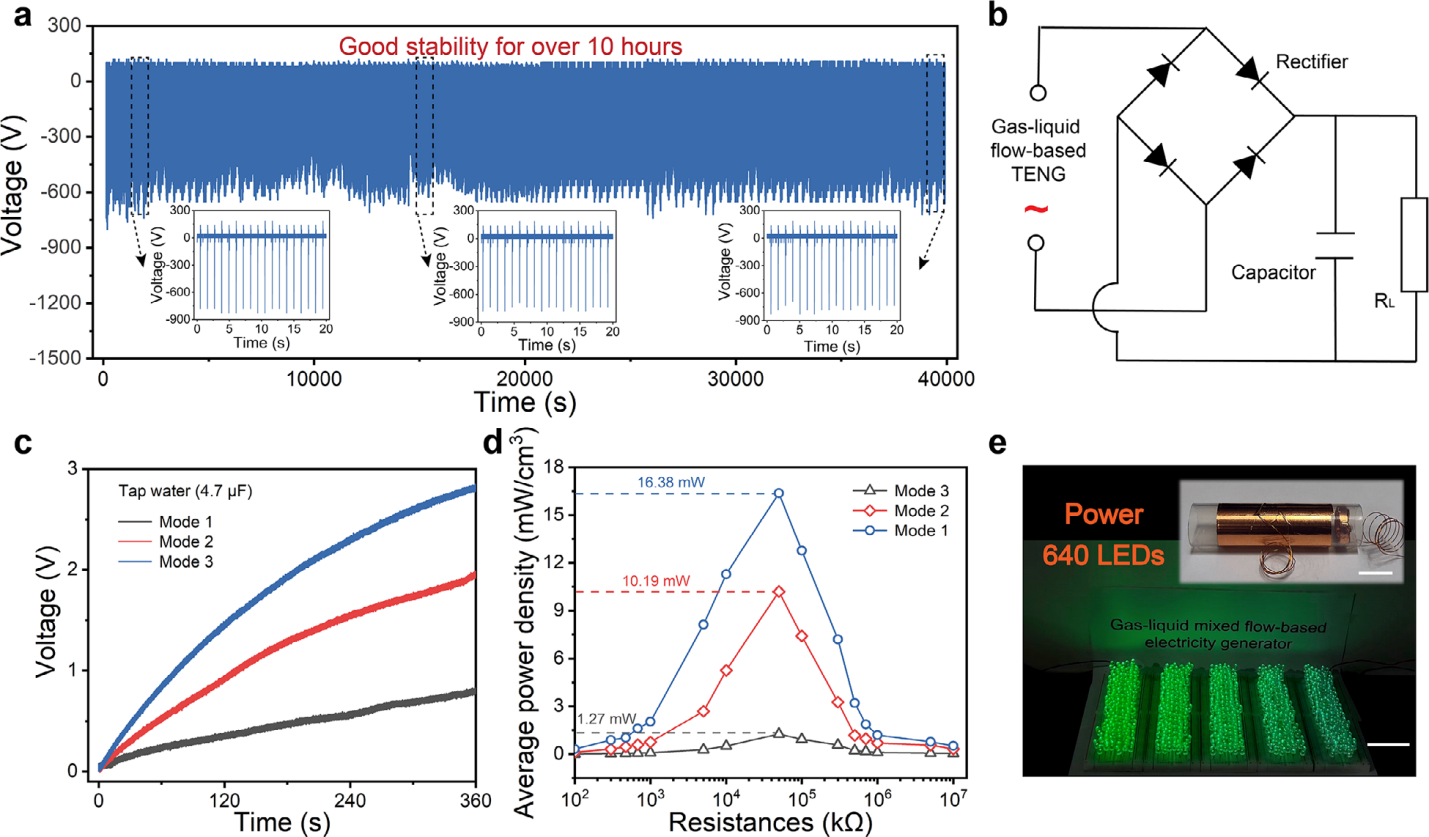
Stabilization and energy harvesting. a) Output voltage stability of TBE‐GL‐TENG. b) Schematic diagram of the rectifier circuit of TBE‐GL‐TENG for energy harvesting. c) Charging the capacitor of 4.7 µF. d) Average power density when the external resistance changes from 1 × 10^2^ to 1 × 10^7^ kΩ. (e) The photograph of 640 LEDs being powered by a TBE‐GL‐TENG device. The insert shows the photograph of a “Mode 3” device. Scale bars in e) and the insert image are 1 cm.

As mentioned above, compared to the traditional interface effect, the bulk effect can significantly enhance the electrical output performance of GL‐TENGs. The “bulk effect” generators improve electrical output performance by utilizing the overall dielectric properties of liquids (rather than relying solely on the EDL at the solid/liquid interfaces), thereby overcoming the shielding effect limitations of traditional TENGs.^[^ ^23^, ^44^ ^]^ Its core advantages lie in its good stability, high‐voltage triboelectricity, and broad environmental adaptability, offering promising applications in the field of self‐powered high‐voltage energy sources, such as contact‐electro‐catalysis, electro‐responsive soft robots, and other fields. For example, Cao et al. used contact electrification to achieve the methanol‐to‐formaldehyde oxidation in both aqueous and nonaqueous CE‐Chemistry for the first time.^[^ ^45^ ^]^ Remarkably, the formaldehyde production rate in dimethyl sulfoxide was 25 times higher than in aqueous systems, which has already surpassed some photocatalytic processes. Recently, Zheng et al. developed a high‐performance triboelectric bionic robot system with high voltage and low current characteristics, which can generate four channels of 18 kV DC high‐voltage to drive the locomotion of the leech‐inspired soft robot.^[^ ^46^ ^]^ All of these latest research findings indicate that the high‐voltage TBE‐GL‐TENG have broad application potential. Given the importance of the stability of enhanced electrical output by the bulk effect for energy harvesting applications, the stability was subsequently tested using DI water. Prior to the stability test, the PTFE was subjected to ultrasonic treatment with deionized water and ethanol to remove surface contaminants. Subsequently, an ion fan was used to neutralize the charges on the PTFE surface. The gas‐liquid mixing flow maintained a specific pulse frequency with a continuous jet‐on time of 1.0 s and a jet‐off time of 0.1 s, and each pulse cycle containing 1.0 mL of water. As shown in **Figure** **6**a, the output voltage of a TBE‐GL‐TENG with a 6.0‐cm‐long and 1.0‐mm‐thick PTFE tube remains ≈−600 V, demonstrating excellent stability (exceeding 10 h).

To efficiently utilize the generated electrical power, a rectifier circuit was established between the electricity generators and an external load (such as a capacitor) for electric energy storage (Figure 6b). And the rectified DC power output can directly charge the capacitor. As shown in Figure 6c, under the same conditions, three different modes of generators were used to charge a 4.7 µF capacitor. The charging voltage curve showed that “Mode 3” could charge the voltage to ≈3 V in 1 min, which was more efficient than “Mode 1” and “Mode 2.” The output instantaneous power in TBE‐GL‐TENG can be calculated as *P* = *I_L_^2^R*_L_, where *R*_L_ and *I*_L_ are the load impedance and load current, respectively. Under a fixed *R*_L_ and TBE‐GL‐TRNG configuration, *P* is mainly determined by *R*_L_ and *I*_L_, where *R*_L_ also regulates the load current *I*_L_. A maximum *P* of 18.67 mW was obtained at *R*_L_ = 50 MΩ, corresponding to a power density of 18.67 kW m^−3^. This indicates that for a “Mode 3” generator system, 1.0 mL of water contained in the gas‐liquid mixed flow can generate 18.67 mW of electrical power. In addition, after the electrical output performance stabilized, multiple points were selected for calculation to obtain the average power density (Figure 6d). Since the power generation mechanisms are different, there are significant differences in the short‐circuit current and charge transfer between the three power generation modes (Figure S12, Supporting Information). As can be seen from the figure, under the same conditions, the output power of “Mode 3” is much higher than that of “Mode 1” and “Mode 2.” In the condition of *R*_L_ = 50 MΩ, the average power density of the electricity generators “Mode 1,” “Mode 2,” and “Mode 3” were 1.27, 10.19 and 16.38 kW m^−3^ respectively. To demonstrate the practical application capability of TBE‐GL‐TENG, energy harvesting experiments were conducted using readily available and inexpensive tap water. As shown in Figure 6e, the electrical power output of a TBE‐GL‐TENG device can instantaneously light up 640 LEDs.

## 3 Conclusion

In conclusion, to address the problem of relatively low output power density imposed by the traditional interface effect, a novel tubular bulk effect gas‐liquid mixing flow triboelectric nanogenerator (TBE‐GL‐TENG) was designed. The introduction of the electrode inside the PTFE tube successfully coupled the bulk effect, significantly enhancing the instantaneous output performance. The TBE‐GL‐TENG could generate an output voltage of 1530 V and an output current of 112 µA, and the instantaneous power density could reach 18.67 kW m^−3^, which far exceeded the control groups based on the interface effect. The working mechanism of TBE‐GL‐TENG was systematically elucidated from the perspectives of gas‐liquid mixing flow dynamics, theoretical simulation, equivalent circuit, and experiments. And the electrical output of the TBE‐GL‐TENG can be regulated by adjusting key factors such as the length of the PTFE tube, ion concentration, liquid volume, and external load. A single TBE‐GL‐TENG with 1.0 mL tap water could directly power 640 LEDs. This work provides guidance for water energy harvesting and application scenarios of the GL‐TENGs with enhanced electrical output based on the bulk effect.

## 4 Experimental Section

### Materials

Polytetrafluoroethylene (PTFE) tubes with thicknesses of 1.0, 1.5, and 2.0 mm were purchased from Yangzhong Fuda Insulation Electric Co. Ltd. Copper electrodes with a thickness of 60 µm and wires with a diameter of 0.2 mm were purchased from Yueqing Zhiyou Trading Co. Ltd. NaCl (99.5%) and ethanol (99.7%) were obtained from Tianjin Dingshengxin Chemical Industry Co. Ltd. Deionized water (18.2 MΩ.cm) was obtained from the laboratory ultra‐pure water system (Milli‐Q Integral 3 ZRXQ003T0). NaCl aqueous solutions of different concentration gradients were prepared using high‐purity NaCl granules and DI water. A high‐precision digital peristaltic pump for controlling water flow was purchased from Nanjing Runze Fluid Control Equipment Co. Ltd.

### Characterization

The surface morphology of PTFE was observed using an electron microscope. The static contact angle (CA) between water and the PTFE surface was measured using a DSA‐100 optical contact angle meter (Kruss Company, Ltd., Germany). The kinetic behavior of liquids in tubular gas‐liquid mixed flow was recorded by a high‐speed camera (Revealer) at a typical recording speed of 40 000 frames per second. The conductivity and TDS values of the NaCl solution were obtained using a Delixi Electrical Conductivity Tester (Table S2, Supporting Information).

### Electrical Measurement

The output voltage of the power generation devices was obtained by utilizing a portable mixed signal oscilloscope (Tektronix, 2 Series MSO) equipped with a high‐impedance (100 megohms) probe. The output current of was measured by a low‐noise current preamplifier (SR570, Stanford Research System, America) connected to the oscilloscope. The Filter Type of SR570 and the bandwidth of the oscilloscope were set at “NONE” and 100 MHz to obtained an electric signal with high fidelity. Data were collected and analyzed through LabVIEW Based Development System (National Instruments). All experiments were performed at room temperature.

### Fabrication of the TBE‐GL‐TENG

The TBE‐GL‐TENG mainly consists of a PTFE tube, an annular electrode on the outer surface of the tube, an annular electrode on the inner surface of the tube, and several connecting wires. First, the PTFE tube was cut to the desired lengths of 2, 4, 6, 8, and 10 cm. It was then sequentially immersed in deionized water and ethanol for ultrasonic treatment for 5 min to remove surface contaminants. After drying, the Cu electrodes were attached to the outer wall of the tube as the negative electrode, and Cu electrode were attached to the inner wall as the positive electrode. Finally, the positive and negative electrodes are connected via an external circuit to form a complete TBE‐GL‐TENG. Similar methods are used to prepare the traditional control group power generation devices “Mode 1” and “Mode 2.”

### Theoretical Simulation

The voltage distribution at the water‐PTFE interface was simulated using the electrostatic field module in COMSOL Multiphysics software. A 2D physical model of the water‐PTFE interface was created, followed by mesh generation and boundary condition setup for voltage simulation. The simulation results represent the static contact between water and PTFE (Figure 2d). The surface potential distributions for “Mode 1,” “Mode 2,” and “Mode 3” devices were simulated using the COMSOL Multiphysics software, as shown in Figure 3b,e,h. The simulated region size was 30 µm × 30 µm, with connected regions simulated using the circuit module. The surface charge density of the droplet was set to 3e^−4^ C m^−^^2^, and that of PTFE was set to −3e^−4^ C m^−^^2^. The outer small boundary was set as an infinite element domain, indicating an externally infinite domain. The infinite element domain boundary was set to 0 V, and the grounded end was set to 0 V. To simulate the transfer charge between PTFE and water, a 3D physical model of PTFE and a series of water with varying NaCl concentration gradients was established. All computations are performed within the framework of density functional theory (DFT) as implemented in the Vienna Ab initio Simulation Package code by using the projector augmented wave method with the Perdew‐Burke‐Ernzerhof exchange‐correlation functional,^[^ ^47^, ^48^ ^]^ and the plane wave energy cutoff was set to 500 eV. To simplify the calculations, ten water molecules were simulated with 0, 1, 2, 3, 4, 5, and 6 NaCl ion clusters to simulate the Bader charges between PTFE and water. DFT simulation results indicate that as the concentration of NaCl ions in water increases moderately, the charge transfer between PTFE and water gradually increases (Figure 5e). This was consistent with the results of previous experiments.

## Conflict of Interest

The authors declare no conflict of interest.

## Author Contributions

Y.D. and W.L. conceived the idea. Y.D. designed the experiment, analyzed the data, and wrote the paper. W.L. and N.W. supervised the research. J.C. helped fabricate the device. D.Y. and Z.C. designed the materials of the device. S.C. helped with the experiments. All the authors discussed the results and commented on the manuscript.

## Supporting information



Supporting Information

Supplemental Video 1

## Data Availability

The data that support the findings of this study are available from the corresponding author upon reasonable request.

## References

[1] C. Zhang , Y. Hao , X. Lu , W. Su , H. Zhang , Z. L. Wang , X. Li , Nano‐Micro Lett. 2025, 17, 124. 10.1007/s40820-024-01640-w PMC11785903 39888455

[2] Y. Cao , S. Fan , Y. Tang , Q. Shan , C. Gao , N. Sepúlveda , D. Hou , G. Zhang , Cell Rep. Phys. Sci. 2024, 5, 102117.

[3] Y. Ding , L. Deng , X.‐X. Liu , L. Wang , Y. Meng , M. Ouyang , F. Chen , Z.‐X. Huang , T. Kuang , Chem. Eng. J. 2025, 516, 164101.

[4] Q. Shao , Y. Yi , C. Li , J. Liu , Y. Xie , Q. Gong , Z. Liu , Y. Chen , Renew. Energy 2025, 253, 123590.

[5] A. M. Alinia , M. Sheikholeslami , Sci. Rep. 2025, 15, 1336. 39779786 10.1038/s41598-025-85161-5 PMC11711203

[6] J. Han , S. H. Park , Y. S. Jung , Y. S. Cho , Nat. Commun. 2024, 15, 4129. 38755193 10.1038/s41467-024-48551-3 PMC11099020

[7] X. Cao , Y. Xiong , J. Sun , X. Zhu , Q. Sun , Z. L. Wang , Adv. Funct. Mater. 2021, 31, 2102983.

[8] X. Chen , D. Goodnight , Z. Gao , A. H. Cavusoglu , N. Sabharwal , M. DeLay , A. Driks , O. Sahin , Nat. Commun. 2015, 6, 7346. 26079632 10.1038/ncomms8346 PMC4490384

[9] T. Sun , L. Wang , W. Jiang , Mater. Today 2022, 57, 121.

[10] J. Meng , L. Zhang , H. Liu , W. Sun , W. Wang , H. Wang , D. Yang , M. Feng , Y. Feng , D. Wang , Adv. Energy Mater. 2023, 14, 2303298.

[11] X. Li , L. Zhang , Y. Feng , Y. Zhang , H. Xu , F. Zhou , D. Wang , ACS Nano 2023, 17, 23977. 38010973 10.1021/acsnano.3c08742

[12] Y. Qin , Y. Wang , X. Sun , Y. Li , H. Xu , Y. Tan , Y. Li , T. Song , B. Sun , Angew.Chem., Int. Ed. 2020, 59, 10619. 10.1002/anie.202002762 32187779

[13] H. Wu , Z. Wang , Y. Zi , Adv. Energy Mater. 2021, 11, 2100038.

[14] H. Zhao , M. Xu , M. Shu , J. An , W. Ding , X. Liu , S. Wang , C. Zhao , H. Yu , H. Wang , C. Wang , X. Fu , X. Pan , G. Xie , Z. L. Wang , Nat. Commun. 2022, 13, 3325. 35680888 10.1038/s41467-022-31042-8 PMC9184604

[15] F.‐R. Fan , Z.‐Q. Tian , Z. L. Wang , Nano Energy 2012, 1, 328.

[16] Z. H. Lin , G. Cheng , L. Lin , S. Lee , Z. L. Wang , Angew. Chem., Int. Ed. 2013, 52, 12545. 10.1002/anie.201307249 24123530

[17] X. Xu , P. Li , Y. Ding , W. Xu , S. Liu , Z. Zhang , Z. Wang , Z. Yang , Energy Environ. Sci. 2022, 15, 2916.

[18] C. Wang , J. Wang , P. Wang , Y. Sun , W. Ma , X. Li , M. Zhao , D. Zhang , Adv. Mater. 2024, 36, 2400505. 10.1002/adma.202400505 38782490

[19] Z. L. Wang , Nature 2017, 542, 159. 28179678 10.1038/542159a

[20] G. H. Nam , J. H. Ahn , G. H. Lee , C. P. Vo , K. K. Ahn , Adv. Energy Sustain. Res. 2020, 1, 2000031.

[21] G. Xue , Y. Xu , T. Ding , J. Li , J. Yin , W. Fei , Y. Cao , J. Yu , L. Yuan , L. Gong , J. Chen , S. Deng , J. Zhou , W. Guo , Nat. Nanotechnol. 2017, 12, 317. 28135262 10.1038/nnano.2016.300

[22] X. Zhao , Z. Xiong , Z. Qiao , X. Bo , D. Pang , J. Sun , J. Bian , Chem. Eng. J. 2022, 434, 134671.

[23] W. Xu , H. Zheng , Y. Liu , X. Zhou , C. Zhang , Y. Song , X. Deng , M. Leung , Z. Yang , R. X. Xu , Z. L. Wang , X. C. Zeng , Z. Wang , Nature 2020, 578, 392. 32025037 10.1038/s41586-020-1985-6

[24] N. Zhang , H. Gu , K. Lu , S. Ye , W. Xu , H. Zheng , Y. Song , C. Liu , J. Jiao , Z. Wang , X. Zhou , Nano Energy 2021, 82, 105735.

[25] Y. Zheng , T. Liu , J. Wu , T. Xu , X. Wang , X. Han , H. Cui , X. Xu , C. Pan , X. Li , Adv. Mater. 2022, 34, 2202238. 10.1002/adma.202202238 35538660

[26] H. Zhang , G. Dai , Y. Luo , T. Zheng , T. Xiongsong , K. Yin , J. Yang , ACS Energy Lett. 2024, 9, 1431.

[27] B. Zhang , W. Xu , L. Peng , Y. Li , W. Zhang , Z. Wang , Nat. Rev. Electr. Eng. 2024, 1, 218.

[28] K.‐H. Lee , M.‐G. Kim , W. Kang , H.‐M. Park , Y. Cho , J. Hong , T.‐H. Kim , S.‐H. Kim , S.‐K. Cho , D. Kang , S.‐W. Kim , C. Jo , S.‐Y. Lee , Nano‐Micro Lett. 2025, 17, 210. 10.1007/s40820-025-01714-3 PMC11985731 40208459

[29] N. Zhang , H. Zhang , W. Xu , H. Gu , S. Ye , H. Zheng , Y. Song , Z. Wang , X. Zhou , Droplet 2022, 1, 56.

[30] H. Zhang , R. Zhang , Y. Zhao , K. Xiao , Z. Wu , Z. Ding , Y. Yang , J. Li , Adv. Funct. Mater. 2025, 35, 2424446.

[31] L. Li , X. Li , W. Deng , C. Shen , X. Chen , H. Sheng , X. Wang , J. Zhou , J. Li , Y. Zhu , Z. Zhang , J. Yin , W. Guo , Sci. Adv. 2023, 9, adi2993. 10.1126/sciadv.adi2993 PMC10651119 37967189

[32] Y. Dong , N. Wang , D. Yang , J. Wang , W. Lu , D. Wang , Adv. Funct. Mater. 2023, 33, 2300764.

[33] Y. Dong , S. Xu , C. Zhang , L. Zhang , D. Wang , Y. Xie , N. Luo , Y. Feng , N. Wang , M. Feng , X. Zhang , F. Zhou , Z. L. Wang , Sci. Adv. 2022, 8, add0464. 10.1126/sciadv.add0464 PMC9710874 36449611

[34] C. Li , Y. Qin , H. Zhang , Y. Wang , J. Liao , H. Guo , Nano Energy 2024, 120, 109138.

[35] Q. Wu , W. Wang , L. Zhang , X. Wu , X. Zhang , D. Wang , Nano Energy 2024, 126, 109642.

[36] H. Zou , Y. Zhang , L. Guo , P. Wang , X. He , G. Dai , H. Zheng , C. Chen , A. C. Wang , C. Xu , Z. L. Wang , Nat. Commun. 2019, 10, 1427. 30926850 10.1038/s41467-019-09461-x PMC6441076

[37] Z. L. Wang , A. C. Wang , Mater. Today 2019, 30, 34.

[38] P. García‐Sánchez , A. Ramos , Phys. Rev. E 2015, 92, 052313. 10.1103/PhysRevE.92.052313 26651701

[39] M. Sun , Q. Lu , Z. L. Wang , B. Huang , Nat. Commun. 2021, 12, 1752. 33741951 10.1038/s41467-021-22005-6 PMC7979908

[40] Y. Jin , S. Yang , M. Sun , S. Gao , Y. Cheng , C. Wu , Z. Xu , Y. Guo , W. Xu , X. Gao , S. Wang , B. Huang , Z. Wang , Nat. Commun. 2024, 15, 4762. 38834547 10.1038/s41467-024-49088-1 PMC11150272

[41] J. Nie , Z. Ren , L. Xu , S. Lin , F. Zhan , X. Chen , Z. L. Wang , Adv. Mater. 2020, 32, 1905696. 10.1002/adma.201905696 31782572

[42] M. Z. Bazant , K. Thornton , A. Ajdari , Phys. Rev. E 2004, 70, 021506. 10.1103/PhysRevE.70.021506 15447495

[43] L. Collins , S. Jesse , J. I. Kilpatrick , A. Tselev , O. Varenyk , M. B. Okatan , S. A. L. Weber , A. Kumar , N. Balke , S. V. Kalinin , B. J. Rodriguez , Nat. Commun. 2014, 5, 3871. 24846328 10.1038/ncomms4871

[44] C. Cao , Z. Li , F. Shen , Q. Zhang , Y. Gong , H. Guo , Y. Peng , Z. L. Wang , Energy Environ. Sci. 2024, 17, 885.

[45] T. Gan , Z. Yang , S. Li , H. Qian , Z. Li , J. Liu , P. Peng , J. Bai , H. Liu , Z. Wang , D. Wei , J. Am. Chem. Soc. 2025, 147, 25407. 40645920 10.1021/jacs.5c05124

[46] Q. Zheng , L. Xin , Q. Zhang , F. Shen , X. Lu , C. Cao , C. Xin , Y. Zhao , H. Liu , Y. Peng , J. Luo , H. Guo , Z. Li , Adv. Mater. 2025, 37, 2417380. 10.1002/adma.202417380 39775869

[47] G. Kresse , J. Furthmüller , Phys. Rev. B 1996, 54, 11169. 10.1103/physrevb.54.11169 9984901

[48] J. P. Perdew , K. Burke , M. Ernzerhof , Phys. Rev. Lett. 1996, 77, 3865. 10062328 10.1103/PhysRevLett.77.3865

