# Supplementary material for: Enhancing the Output Performance of Tubular Gas‐Liquid Mixing Triboelectric Nanogenerator by Bulk Effect
Source: Adv Sci (Weinh). 2025 Aug 22;12(42):e10787. doi: 10.1002/advs.202510787 (PMC12622509; doi:10.1002/advs.202510787)
Supplement: Supplementary file 1 — Supporting Information [file ADVS-12-e10787-s001.docx]

Supplementary Materials for

**Enhancing the Output Performance of Tubular Gas-liquid Mixing Triboelectric Nanogenerator by Bulk Effect**

Yang Dong ^a, b^, Jiahui Cheng ^a^, Zhichen Cao ^a^, Nannan Wang ^c^, Di Yang ^c^,

Suping Chang ^a^, Wenlong Lu ^b, d *^

^a^ School of Mechanical Science and Engineering, Huazhong University of Science and Technology, Wuhan 430074, PR China.

^b^ State Key Laboratory of Intelligent Manufacturing Equipment and Technology, Huazhong University of Science and Technology, Wuhan, 430074, Hubei.

^c^ State Key Laboratory of Solid Lubrication, Lanzhou Institute of Chemical Physics, Chinese Academy of Sciences, Lanzhou 730000, China.

^d^ HUST-Shenzhen Research Institute, Shenzhen 518000, PR China.

*Corresponding Author E-mail: [hustwenlong@mail.hust.edu.cn](mailto:hustwenlong@mail.hust.edu.cn)

The file includes:

**Figure S1.** Typical time-resolved voltage waveform of a TBE-GL-TENG device.

**Figure S2.** One full current cycle and corresponding charge accumulation curve.

**Figure S3.** Measure the charge characteristics of the droplets after sliding on a PTFE surface.

**Figure S4.** Finite element simulation of “Mode 1” device using COMSOL software.

**Figure S5.** Finite element simulation of “Mode 2” device using COMSOL software.

**Figure S6.** Finite element simulation of “Mode 3” device using COMSOL software.

**Figure S7.** Design of power generator with conventional interfacial effect.

**Figure S8.** Relationship between the output performance of “Mode 1” device and the volume of deionized water.

**Figure S9.** Relationship between the output performance of “Mode 2” device and the volume of deionized water.

**Figure S10.** Simulation of charge transfer at the water/PTFE interface based on density functional theory (DFT).

**Figure S11.** Output performance of “Mode 3” generator versus thickness of PTFE tube.

**Figure S12.** Current and charge transfer in the three power generation modes with a 50 MΩ load.

**Table S1.** The comparison of previous work and our work.

**Table S2.** Physical properties of NaCl solution and tap water.

**Video S1.** (mp4 format). Recording the dynamics of gas-liquid mixed flow by a high-speed camera.





**Figure S1.** Typical time-resolved voltage waveform of a TBE-GL-TENG device. When both the gas phase and liquid phase pass through the TBE-GL-TENG, the electrical output is significantly greater than when only the gas flow passes through (almost undetectable), indicating that compared to the triboelectricity at the water/PTFE interface, the triboelectricity at the gas/PTFE interface can be neglected.


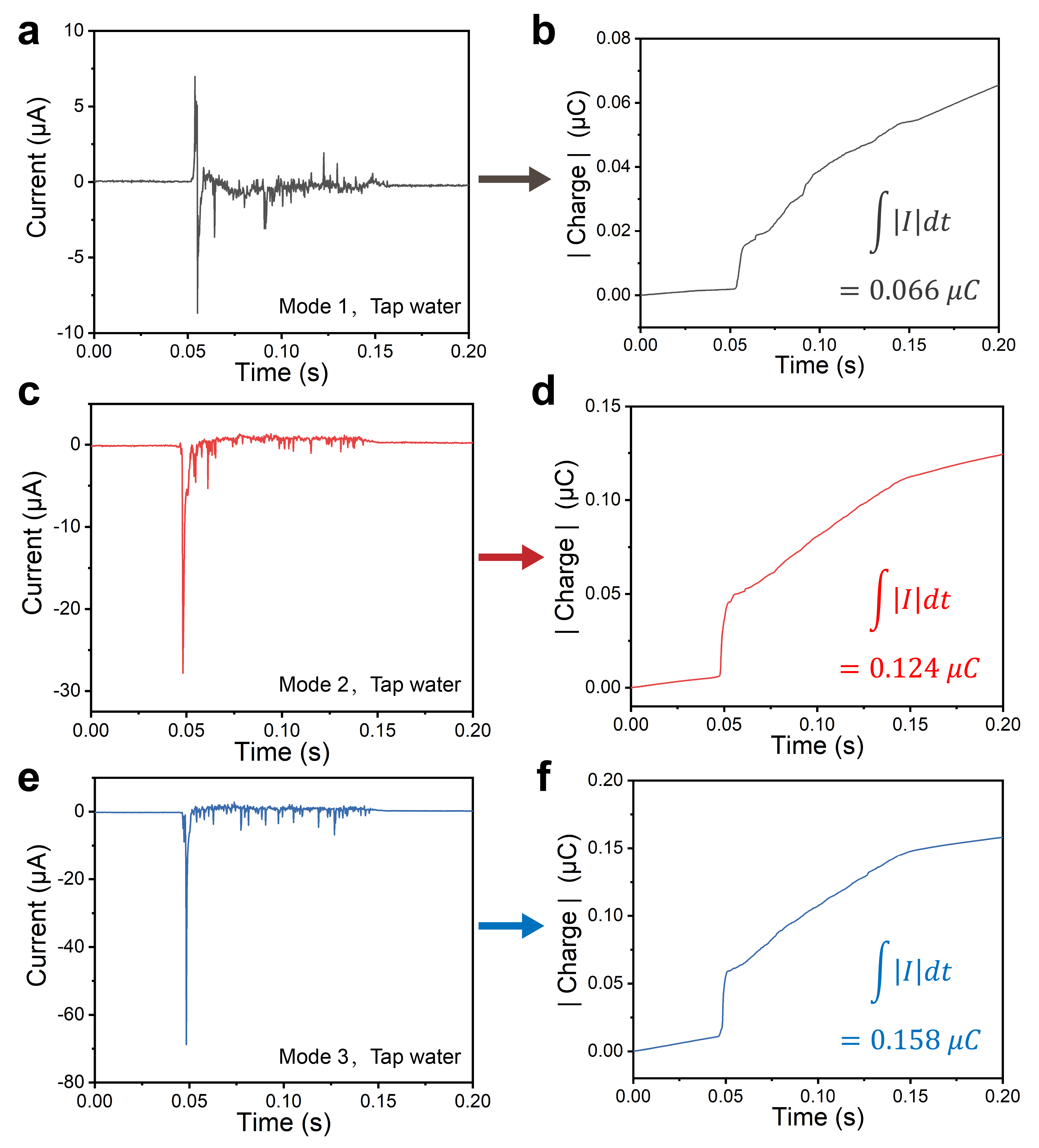


**Figure S2.** One full current cycle and corresponding charge accumulation curve. (a) and (b) are the current and transferred charge in “Mode 1,” respectively. (c) and (d) are the current and transferred charge in “Mode 2,” respectively. (e) and (f) are the current and transferred charge in “Mode 3,” respectively.


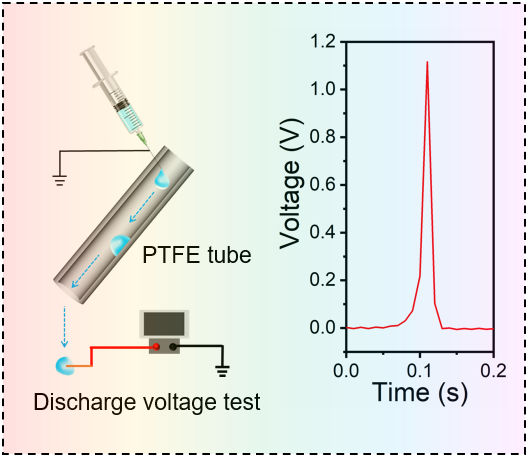


**Figure S3.** Measure the charge characteristics of the droplets after sliding on a PTFE surface. The results showed that a neutrally charged water droplet became positively charged after sliding over a PTFE surface.


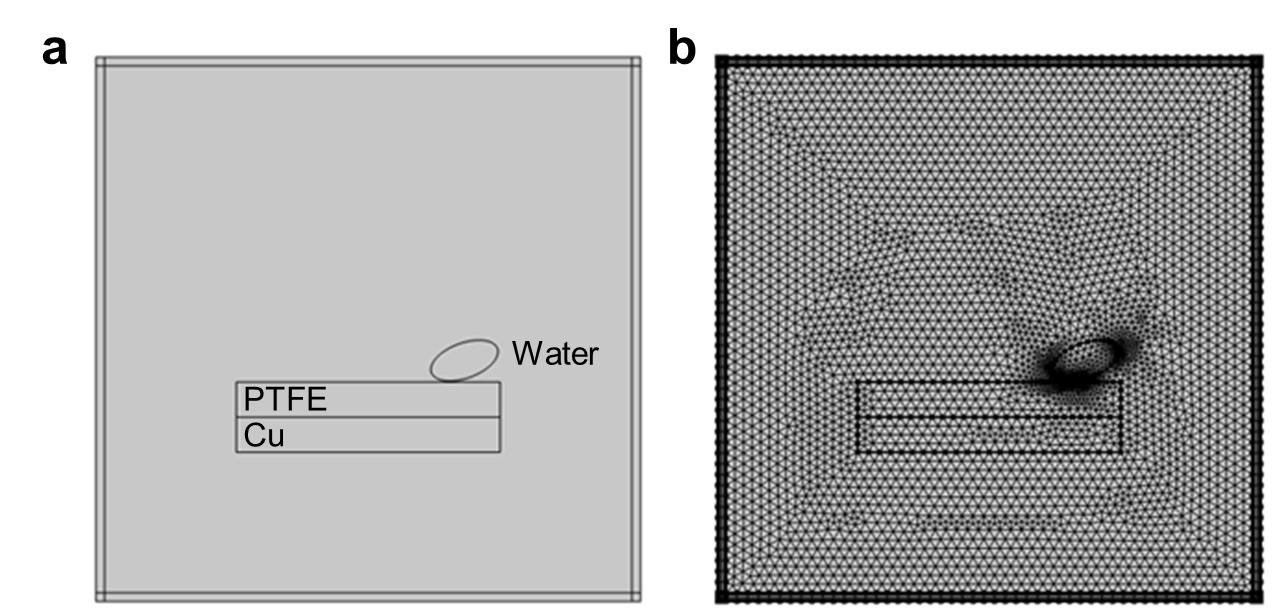


**Figure S4.** Finite element simulation of “Mode 1” device using COMSOL software. (a) Geometric model. (b) Mesh generation. The overall model size was 30 um x 30 um.


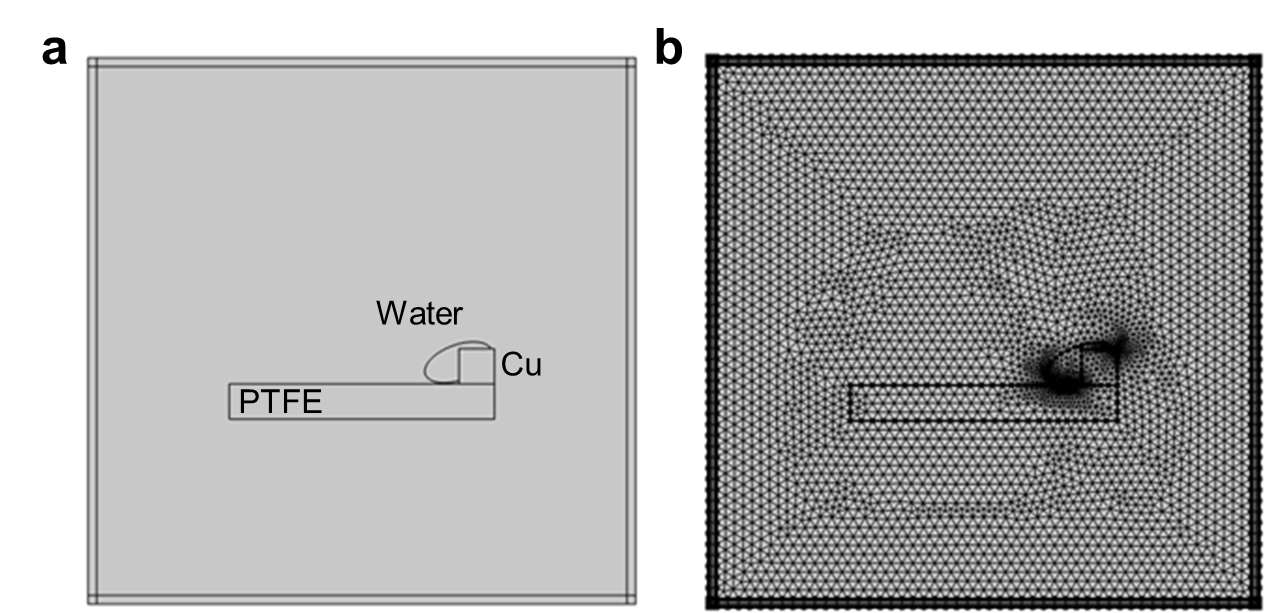


**Figure S5.** Finite element simulation of “Mode 2” device using COMSOL software. (a) Geometric model. (b) Mesh generation. The overall model size was 30 um x 30 um.


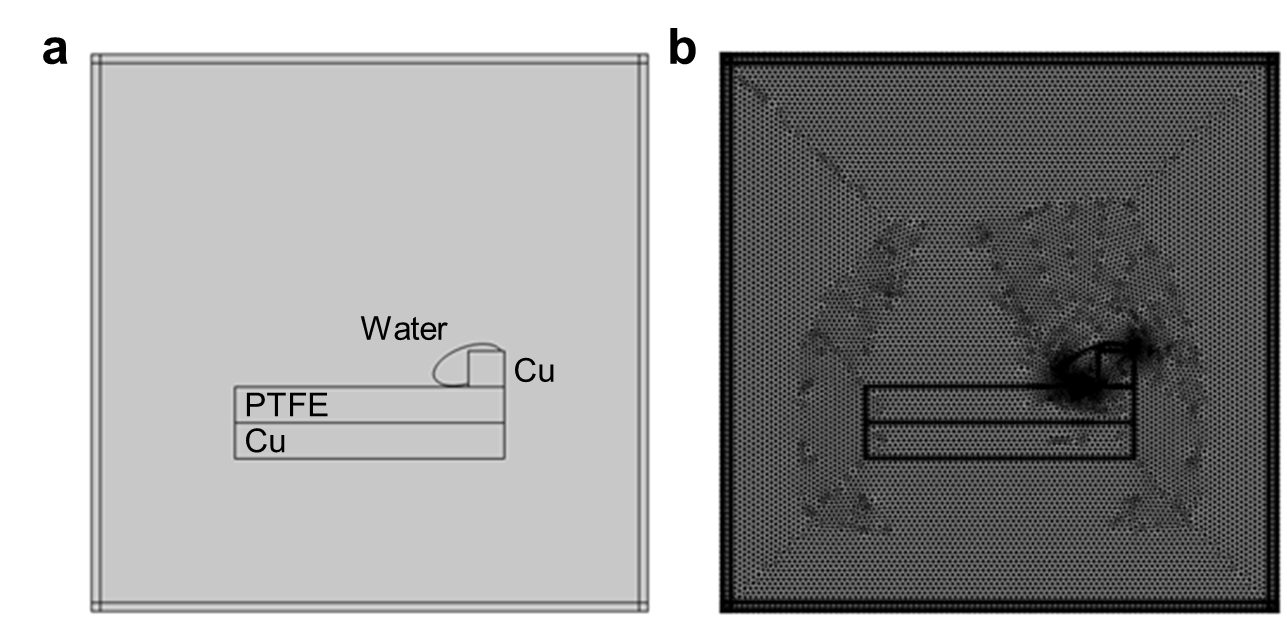


**Figure S6.** Finite element simulation of “Mode 3” device using COMSOL software. (a) Geometric model. (b) Mesh generation. The overall model size was 30 um x 30 um.


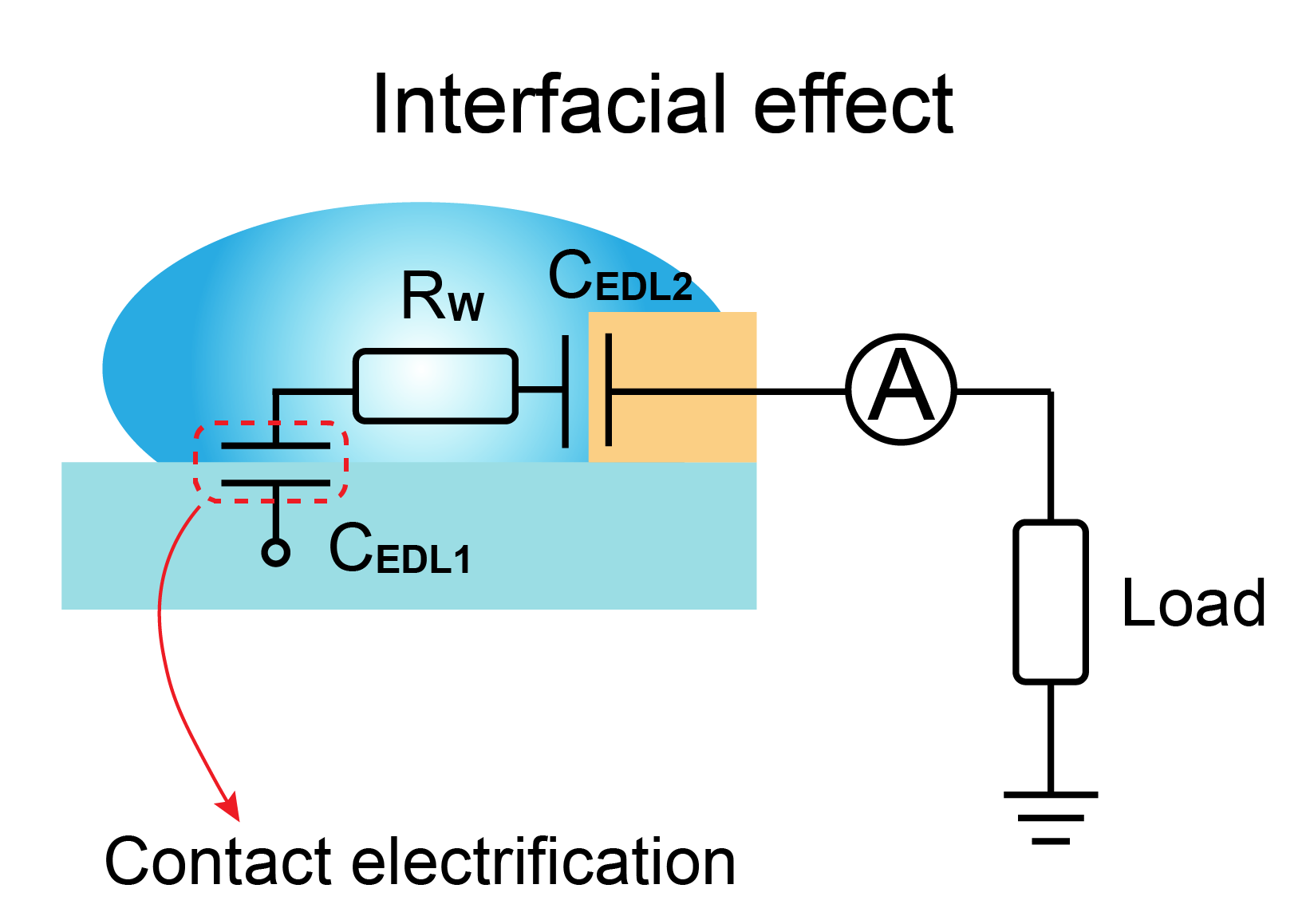


**Figure S7.** Design of power generator with conventional interfacial effect.


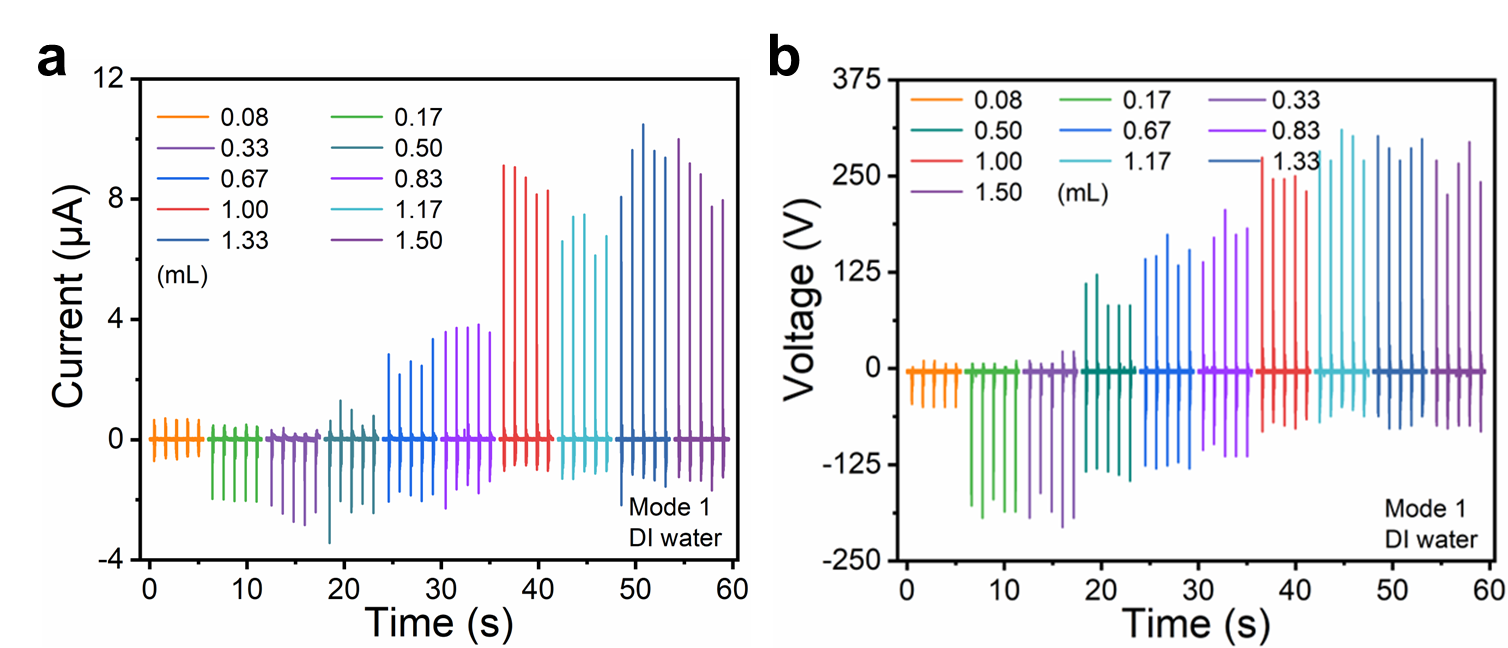


**Figure S8.** Relationship between the output performance of “Mode 1” device and the volume of deionized water.


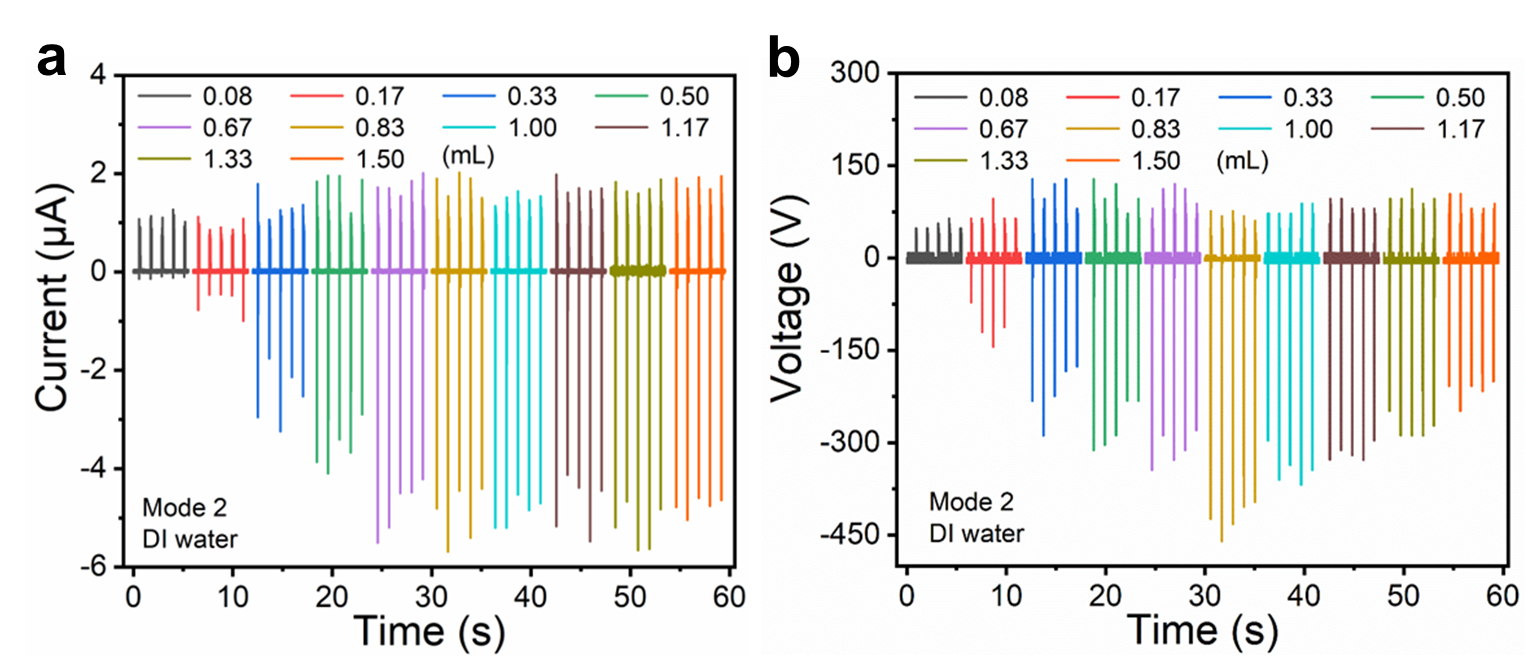


**Figure S9.** Relationship between the output performance of “Mode 2” device and the volume of deionized water.


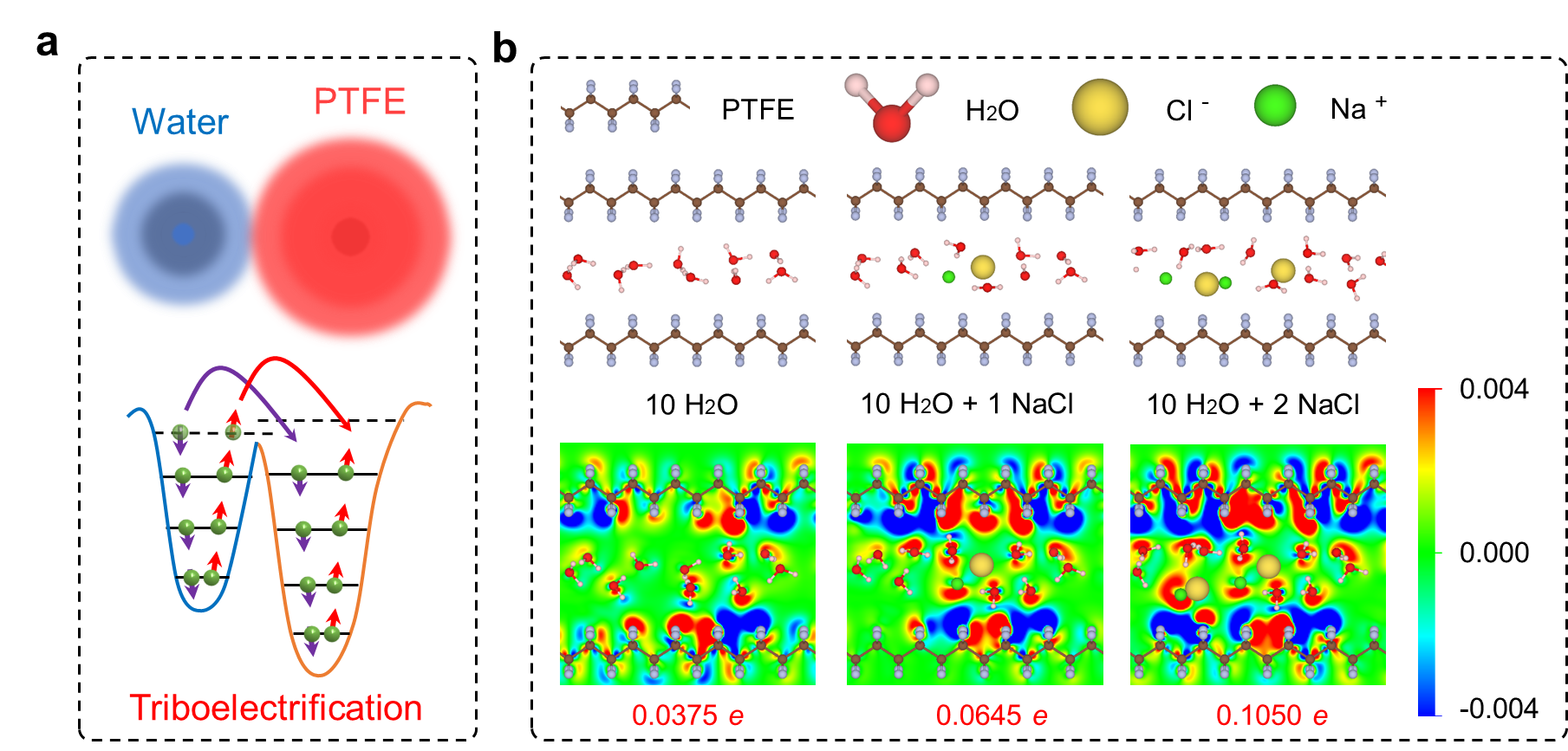


**Figure S10.** Simulation of charge transfer at the water/PTFE interface based on density functional theory (DFT). (a) Schematic diagram of the microscopic model of water/PTFE contact electrification: water loses electrons and becomes positively charged, while PTFE gains electrons and becomes negatively charged. (b) Simulation of charge transfer between water and PTFE, regardless of whether the water contains Na^+^ and Cl^-^ or only a small amount of them. The simulation results showed that the amount of charge transferred between water and PTFE increases with an appropriate increase in ion concentration.


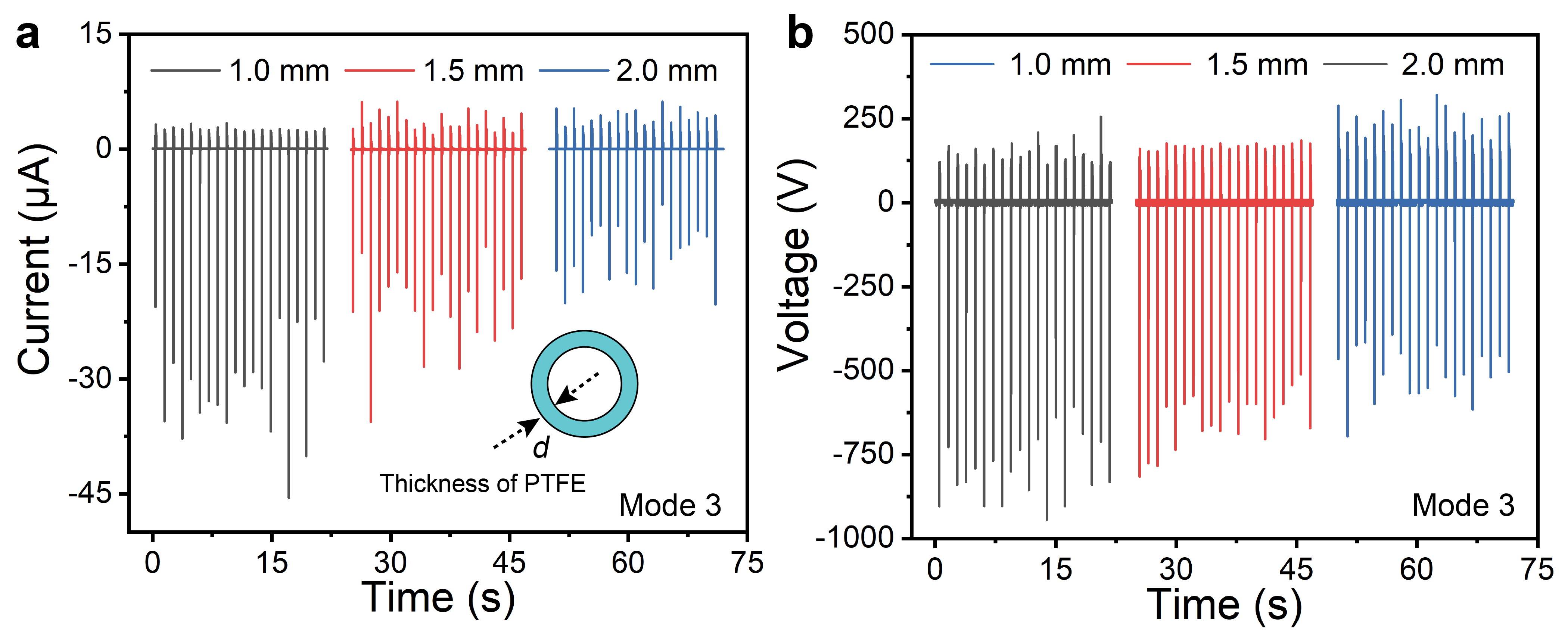


**Figure S11.** Output performance of “Mode 3” generator versus thickness of PTFE tube. (a) Voltage versus thickness of PTFE tube. (b) Current versus thickness of PTFE tube.


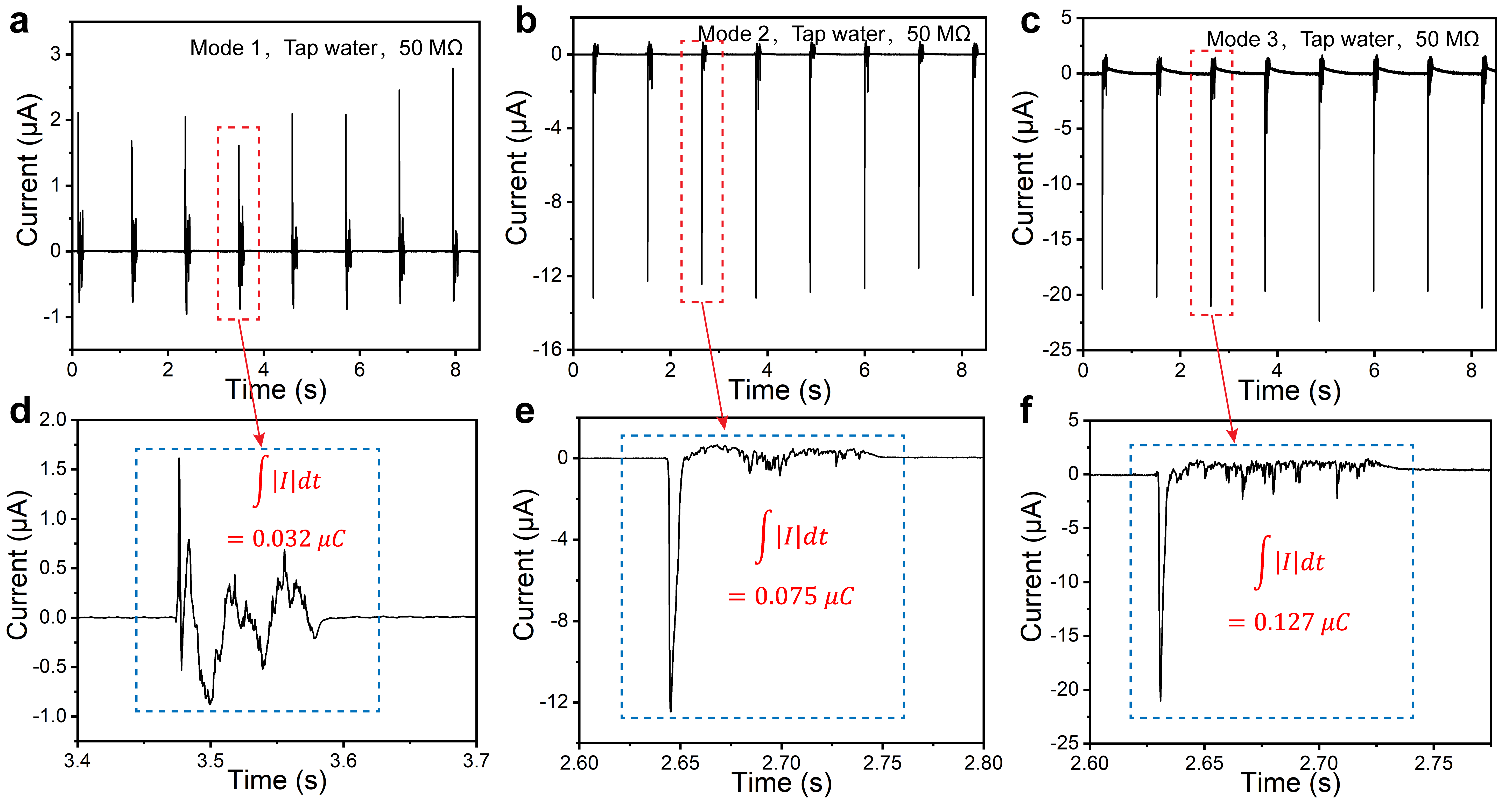


**Figure S12.** Current and charge transfer in the three power generation modes with a 50 MΩ load. (a) - (c) are the short-circuit currents for “Modes 1”, “Mode 2” and “Mode 3”, respectively. (d) – (e) are the corresponding transfer charges.

**Table S1.** The comparison of previous work and our work.

| Device /  Method | Liquid form | | Working principle | Voltage  Value (V) | Current  Value (μA) | Reporting year | Authors |
| --- | --- | --- | --- | --- | --- | --- | --- |
| DEG | Droplet | CE +  Bulk Effect | | 143.5 | 270.0 | 2020 | W. Xu, et al. [ref 23] |
| GL-TENG | Gas-liquid two-phase flow | | CE +  Breakdown effect | 3789.0 | 867.0 | 2022 | Y. Dong, et al. [ref 33] |
| S-TENG | Spray | | Triboelectric effect | 240.0 | 15.0 | 2024 | C. Li, et al. [ref 34] |
| SVE-TLSTENG | Water wave | | Space volume effect | 230.0 | 0.41 | 2024 | H. Zhang, et al. [ref 26] |
| HWF-TENG | Water flow | | CE +  Electrostatic induction effect | 2270.0 | 141.0 | 2024 | Q. Wu, et al. [ref 35] |
| TBE-ENG | Water wave | | CE +  Bulk Effect | 268.3 | 5000.0 | 2025 | H. Zhang, et al. [ref 30] |
| TBE-GL-TENG | **Gas-liquid mixed flow** | | **CE +**  **Bulk Effect** | **1530.0** | **112.0** | **2025** |  |

Note: CE refers to “Contact Electrification”

**Table S2**. Physical properties of NaCl solution and tap water.

| NaCl concentration  (mmol/L) | 0 (DI) | 100 | 200 | 300 | 400 | 500 | 600 | Tap |
| --- | --- | --- | --- | --- | --- | --- | --- | --- |
| Conductivity  (μs/cm) | 0.52 | 10290 | 19570 | 31760 | 40190 | 48760 | 55680 | 443 |
| Total Dissolved Solids (PPT) | 0.0004 | 5.14 | 9.84 | 14.46 | 18.67 | 24.38 | 27.84 | 0.22 |
